# Molecular subtypes explain lupus epigenomic heterogeneity unveiling new regulatory genetic risk variants

**DOI:** 10.1038/s41525-024-00420-0

**Published:** 2024-07-16

**Authors:** Olivia Castellini-Pérez, Elena Povedano, Guillermo Barturen, Manuel Martínez-Bueno, Andrii Iakovliev, Martin Kerick, Raúl López-Domínguez, Concepción Marañón, Javier Martín, Esteban Ballestar, PRECISEADS Clinical Consortium, Lorenzo Beretta, Barbara Vigone, Jacques‐Olivier Pers, Alain Saraux, Valérie Devauchelle‐Pensec, Divi Cornec, Sandrine Jousse‐Joulin, Bernard Lauwerys, Julie Ducreux, Anne‐Lise Maudoux, Carlos Vasconcelos, Ana Tavares, Esmeralda Neves, Raquel Faria, Mariana Brandão, Ana Campar, António Marinho, Fátima Farinha, Isabel Almeida, Miguel Angel Gonzalez‐Gay Mantecón, Ricardo Blanco Alonso, Alfonso Corrales Martínez, Ricard Cervera, Ignasi Rodríguez‐Pintó, Gerard Espinosa, Rik Lories, Ellen De Langhe, Nicolas Hunzelmann, Doreen Belz, Torsten Witte, Niklas Baerlecken, Georg Stummvoll, Michael Zauner, Michaela Lehner, Eduardo Collantes, Rafaela Ortega Castro, Ma Angeles Aguirre‐Zamorano, Alejandro Escudero‐Contreras, Ma Carmen Castro‐Villegas, Norberto Ortego, María Concepción Fernández Roldán, Enrique Raya, Inmaculada Jiménez Moleón, Enrique de Ramon, Isabel Díaz Quintero, Pier Luigi Meroni, Maria Gerosa, Tommaso Schioppo, Carolina Artusi, Carlo Chizzolini, Aleksandra Zuber, Donatienne Wynar, Laszló Kovács, Attila Balog, Magdolna Deák, Márta Bocskai, Sonja Dulic, Gabriella Kádár, Falk Hiepe, Velia Gerl, Silvia Thiel, Manuel Rodriguez Maresca, Antonio López‐Berrio, Rocío Aguilar‐Quesada, Héctor Navarro‐Linares, PRECISEADS Flow Cytometry Study Group, Montserrat Alvarez, Damiana Alvarez‐Errico, Nancy Azevedo, Nuria Barbarroja, Anne Buttgereit, Qingyu Cheng, Carlo Chizzolini, Jonathan Cremer, Aurélie De Groof, Ellen De Langhe, Julie Ducreux, Aleksandra Dufour, Velia Gerl, Maria Hernandez‐Fuentes, Laleh Khodadadi, Katja Kniesch, Tianlu Li, Chary Lopez‐Pedrera, Zuzanna Makowska, Concepción Marañón, Brian Muchmore, Esmeralda Neves, Bénédicte Rouvière, Quentin Simon, Elena Trombetta, Nieves Varela, Torsten Witte, María Orietta Borghi, Weiliang Qiu, Cheng Zhu, Srinivas Shankara, Athina Spiliopoulou, Emanuele de Rinaldis, Elena Carnero-Montoro, Marta E. Alarcón-Riquelme

**Affiliations:** 1 GENYO. Center for Genomics and Oncological Research Pfizer/University of Granada/Andalusian Regional Government, 18016 Granada, Spain; 2 University of Granada, Granada, Spain; 3 Spanish National Research Council (CSIC), Institute of Economy, Geography and Demography, Madrid (IEGD), Madrid, Spain; 4 Autonomous University of Madrid, Madrid, Spain; 5 Department of Genetics, Faculty of Sciences, University of Granada, Granada, Spain; 6 Usher Institute of Population Health Sciences and Informatics. University of Edinburgh Medical School, EH8 9YL Edinburgh, UK; 7 IBPLN-CSIC, Instituto de Parasitología y Biomedicina López-Neyra, Consejo Superior de Investigaciones Científicas, Granada, 18016 Spain; 8 Epigenetics and Immune Disease Group, Josep Carreras Research Institute (IJC), 08916 Badalona, Barcelona Spain; 9 Università degli Studi di Milano and Istituto Auxologico Italiano, Milan, Italy; 10 Sanofi, Early Development and Research, Cambridge, MA USA; 11 Sanofi, Precision Medicine & Computational Biology (PMCB), R&D, Cambridge, MA USA; 12 Institute for Environmental Medicine, Karolinska Institutet, 171 67 Solna, Sweden; 13 Referral Center for Systemic Autoimmune Diseases, Fondazione IRCCS Ca’ Granda Ospedale Maggiore Policlinico di Milano, Milano, Italy; 14 Centre Hospitalier Universitaire de Brest, Hospital de la Cavale Blanche, Brest, France; 15 Pôle de pathologies rhumatismales systémiques et inflammatoires, Institut de Recherche Expérimentale et Clinique, Université catholique de Louvain, Brussels, Belgium; 16 Centro Hospitalar do Porto, Porto, Portugal; 17 Servicio Cantabro de Salud, Hospital Universitario Marqués de Valdecilla, S, antander, Spain; 18 Hospital Clinic I Provicia, Institut d’Investigacions Biomèdiques August Pi i Sunyer, Barcelona, Spain; 19 Katholieke Universiteit Leuven, Leuven, Belgium; 20 Klinikum der Universitaet zu Koeln, Cologne, Germany; 21 Medizinische Hochschule Hannover, Hannover, Germany; 22 Medical University Vienna, Vienna, Austria; 23 Servicio Andaluz de Salud, Hospital Universitario Reina Sofía Córdoba, Córdoba, Spain; 24 Servicio Andaluz de Salud, Complejo hospitalario Universitario de Granada (Hospital Universitario San Cecilio), Granada, Spain; 25 Servicio Andaluz de Salud, Complejo hospitalario Universitario de Granada (Hospital Virgen de las Nieves), Granada, Spain; 26 Servicio Andaluz de Salud, Hospital Regional Universitario de Málaga, Málaga, Spain; 27 Università degli studi di Milano, Milan, Italy; 28 Hospitaux Universitaires de Genève, Genève, Switzerland; 29 University of Szeged, Szeged, Hungary; 30 Charite, Berlin, Germany; 31 Andalusian Public Health System Biobank, Granada, Spain; 32 Immunology and Allergy, University Hospital and School of Medicine, Geneva, Switzerland; 33 Chromatin and Disease Group, Bellvitge Biomedical Research Institute (IDIBELL), Barcelona, Spain; 34 Serviço de Imunologia EX-CICAP, Centro Hospitalar e Universitário do Porto, Porto, Portugal; 35 IMIBIC, Reina Sofia Hospital, University of Cordoba, Córdoba, Spain; 36 Bayer AG, Berlin, Germany; 37 Pharmaceuticals Division, Bayer Pharma, Berlin, Germany; 38 Department of Rheumatology and Clinical Immunology, Charité University Hospital, Berlin, Germany; 39 Department of Microbiology and Immunology, Laboratory of Clinical Immunology, KU Leuven, Leuven, Belgium; 40 Pôle de Pathologies Rhumatismales Inflammatoires et Systémiques, Institut de Recherche Expérimentale et Clinique, Université Catholique de Louvain, Brussels, Belgium; 41 University Hospitals Leuven and Skeletal Biology and Engineering Research Center, KU Leuven, Leuven, Belgium; 42 UCB, Slough, UK; 43 Klinik für Immunologie Und Rheumatologie, Medical University Hannover, Hannover, Germany; 44 NSERM, UMR1227, CHRU Morvan, Lymphocytes B et Autoimmunité, University of Brest, BP 824, Brest, France; 45 Laboratorio di Analisi Chimico Cliniche e Microbiologia - Servizio di Citofluorimetria, Fondazione IRCCS Ca’ Granda Ospedale Maggiore Policlinico di Milano, Milan, Italy

**Keywords:** DNA methylation, Molecular medicine

## Abstract

The heterogeneity of systemic lupus erythematosus (SLE) can be explained by epigenetic alterations that disrupt transcriptional programs mediating environmental and genetic risk. This study evaluated the epigenetic contribution to SLE heterogeneity considering molecular and serological subtypes, genetics and transcriptional status, followed by drug target discovery. We performed a stratified epigenome-wide association studies of whole blood DNA methylation from 213 SLE patients and 221 controls. Methylation quantitative trait loci analyses, cytokine and transcription factor activity - epigenetic associations and methylation-expression correlations were conducted. New drug targets were searched for based on differentially methylated genes. In a stratified approach, a total of 974 differential methylation CpG sites with dependency on molecular subtypes and autoantibody profiles were found. Mediation analyses suggested that SLE-associated SNPs in the HLA region exert their risk through DNA methylation changes. Novel genetic variants regulating DNAm in disease or in specific molecular contexts were identified. The epigenetic landscapes showed strong association with transcription factor activity and cytokine levels, conditioned by the molecular context. Epigenetic signals were enriched in known and novel drug targets for SLE. This study reveals possible genetic drivers and consequences of epigenetic variability on SLE heterogeneity and disentangles the DNAm mediation role on SLE genetic risk and novel disease-specific meQTLs. Finally, novel targets for drug development were discovered.

## Introduction

Systemic lupus erythematosus (SLE) is a systemic autoimmune disease (SAD) caused by the activation of autoreactive T and B cells, the release of inflammatory cytokines and the formation of immune complexes that deposit in tissues, resulting in organ damage. It predominantly appears in young to middle-aged women with a 9:1 female:male bias^1^. Treating and diagnosing SLE is challenged by the patients’ heterogeneity in terms of diversity of symptoms, manifestations^2^, the organs affected, and a diverse array of autoantibody (AAb) specificities. Although AAb production helps in the diagnosis of some autoimmune diseases and is related to several clinical manifestations, the complexity of SLE is such that patients can present a wide range of specificities, being, moreover, not solely identified in SLE or in specific SLE manifestations.

The understanding of genetic, environmental, and molecular mechanisms disrupting immunity and triggering autoantibody production is not well understood. Genome-wide association studies (GWAS) identified several susceptibility genes among which HLA class II locus and genes such as *TNIP, BANK1*, and *IRF5* exhibit the strongest risk effects^3,4^. A strong HLA genetic association with the presence of autoantibodies such as anti-SSB, anti-SSA, anti-RNP, anti-SM^5^, and anti-dsDNA production has been recognized since long^5,6^. The most important molecular characteristic of SLE is the overexpression of several interferon-regulated genes (IRG) known as the interferon (IFN) signature, also widely observed at the epigenetic level (epigenIFNsig) in all blood cell types and tissues^7^ as well as in other SADS^8,9^. A strong interaction between HLA genetic variation, the production of anti-SSA AAb and the epigenIFNsig has been reported in primary Sjogren’s syndrome^9^, however the genetic and autoantibody determinants of the IFN signature in SLE are still not clear. Recently, the study by *Barturen* et al.^8^ molecularly reclassified SLE and other six SADs into different molecular subtypes with important clinical implications. SLE and SADs patients could be stratified into an inflammatory subtype, with increased activity of genes related to the function of monocyte and neutrophil; a lymphoid subtype, with genes related to the function of these immune cells; and an IFN subtype, defined by enhanced activity in genes induced by IFN. This molecular classification needs to be taking into account to overcome SLE heterogeneity.

Heritability studies showed that genetics explains only a small fraction, <10% of SLE susceptibility^10–12^, suggesting the important contribution of environmental or non-genetic factors^13^ and possibly the role of epigenetics mediating gene-by-environment interactions. Epigenetic modifications allow to retrieve different phenotypes from a unique genotype, and are fundamental for immune cells to exhibit diverse and plastic functions responding to evolving environments, stimuli and differentiation processes^14^. Dysregulation at the epigenetic level has been identified in association with SLE and some SLE manifestations^15–17^. The genome of SLE patients is globally hypomethylated -unmethylated state of CpGs in a normally methylated sequence - and a large percentage of SLE patients exhibit the epigenIFNsig but, intriguingly, not all of them. Functional genomics deciphers the regulatory role of many disease-associated non-coding genetic variants^18^, but studies are scarce in SLE^17^. Approaches such as methylation quantitative trait loci (meQTL)^18^ allows identifying genetic variants influencing disease by modifying DNA methylation (DNAm)^17^. However, large meQTL studies have been performed in non-disease populations despite the increasing recognition that regulatory genetic effects can be dependent on age, context, and pathological status^9,18,19^.

Despite the increasing number of molecular and epigenetic studies in SLE, previous work has not paid attention to disease heterogeneity nor to the possible role of genetic factors or their consequences at the transcriptional and cytokines levels.

The present work moves a step forward in the understanding of epigenetic landscapes in SLE, and their possible drivers and consequences, as well as in the identification of potential new SLE drug targets. We speculate that stratifying epigenome-wide association studies (EWAS) based on molecular subtypes and autoantibody specificities, and integrating different multi-omics layers, provides greater statistical power to find new and group-dependent epigenetic associations that might have a specific genetic regulation and context-specific epigenetic correlation with transcriptional factors and cytokine expression.

## Results

**Fig. 1:**
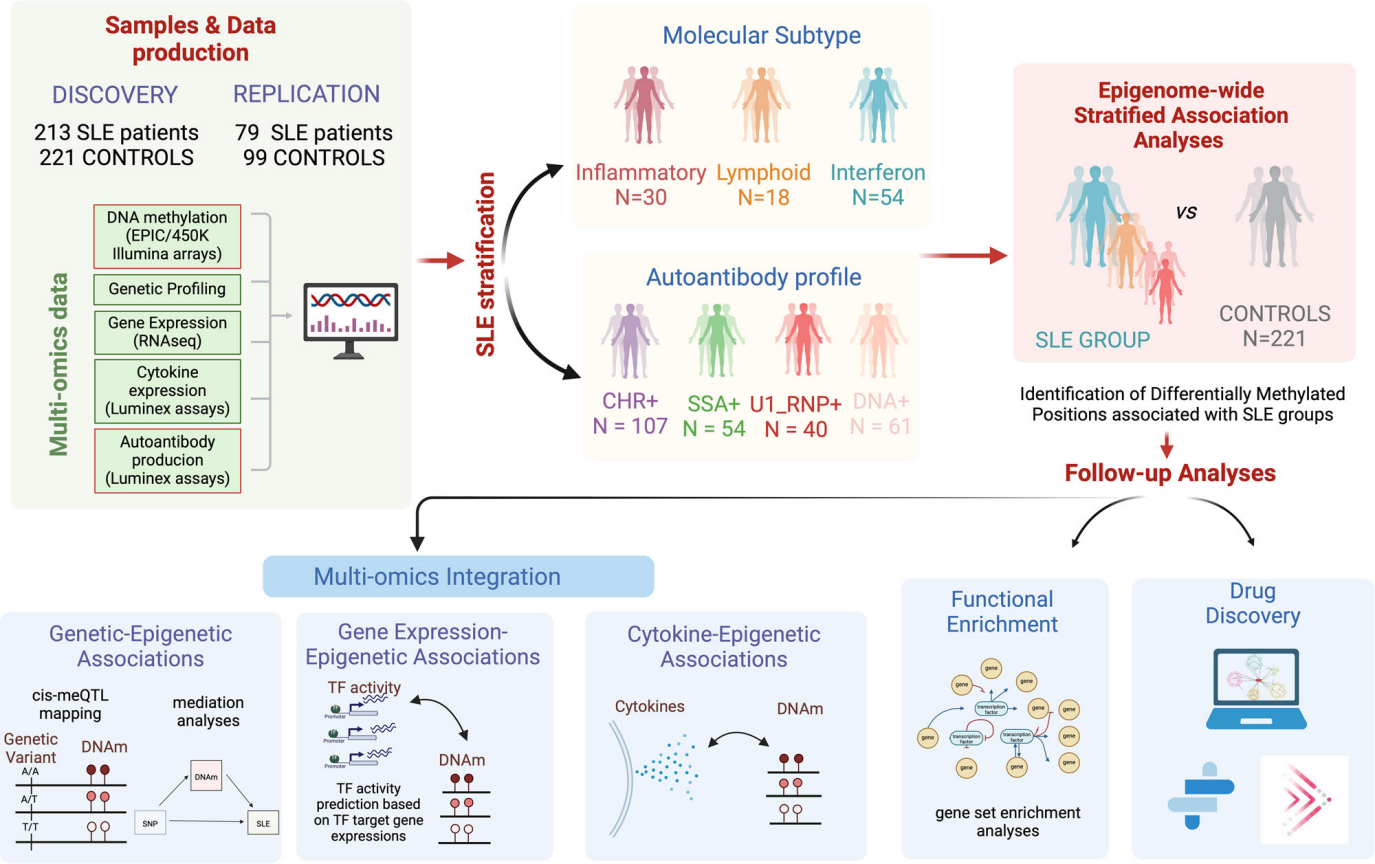
Overview of the study design. Schematic outline illustrating the data analysis and the use of patients’ genetic and molecular information to stratify patients into molecularly homogenous groups and autoantibody-positivity profiles to further perform EWAS functional enrichment analyses, meQTL analyses, cytokine association, and drug discovery according to molecular and serological subtypes.

An overview of the study design is depicted and described in Fig. 1 and Supplementary Table 1.

### Genome-wide DNAm patterns associated with different molecular SLE subtypes

**Fig. 2:**
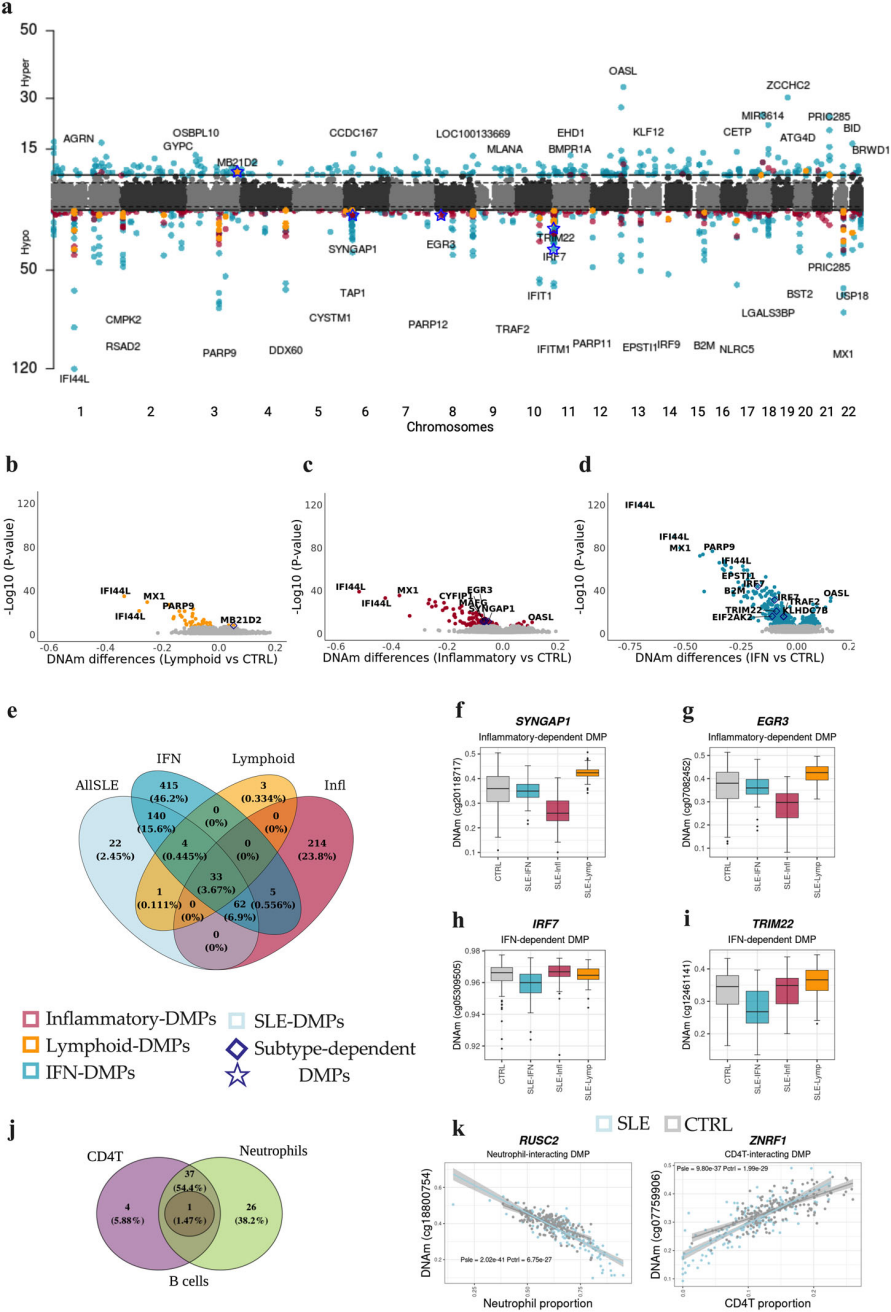
Epigenetic signatures of SLE molecular subtypes and cell type interactions. **a** Manhattan plot illustrating EWAS results for SLE and different molecular groups when compared with controls. X-axis represents the chromosomic locations of CpG sites and Y-axis represents the log10 (*P*) obtained in linear regression models. **b**–**d** Volcano Plots representing EWAS results. The X-axis represents the DNAm differences between each pair of groups tested. **e** Overlap of genome-wide significant results for each EWAS was performed. **f**–**i** Examples of subtype dependent-DMPs. Colored dots represent significant DMPs after Bonferroni correction of different groups according to the legend. Diamonds and starts dots represents subtypes-dependent DMPs. **j** Venn diagram illustrating the overlap of cell-type-interacting DMPs that remain significant after Bonferroni correction. **k** Scatter plots depicting DNAm changes between SLE and CTRL individuals at a specific CpG site identified as a significant DMP in the cell-type-interacting analysis. Neutrophil proportions are represented on the y-axis in the left plot, while CD4T proportions are depicted on the x-axis in the right plot.

We conducted an EWAS with a sample size of 213 SLE cases and 221 controls. This analysis led to the identification of 262 SLE-DMPs (97% replication rate in an independent 450 K sample), as depicted in Fig. 2a and detailed in Supplementary Data. Of these DMPs, 64% displayed hypomethylation effects, while the remaining 36% exhibited hypermethylation effects. Not unexpectedly, the top SLE-DMPs implicated large reductions of DNAm at IRGs and genes enriched in IFN pathways (Supplementary Table 2), as for example *IFI44L*, *MX1*, *NLRC5*, *IFITM1*, *IFIT1*, *IRF9* or *PARP9* but also genes involved in antigen processing and class I presentation (*TAP1* and *B2M*). Large hypermethylation effects were also observed in IRGs such as *OASL*. These results corroborate previous findings and is likely a consequence of the higher IFN levels observed in SLE patients^20^.

Stratifying SLE patients into the molecular subtypes (*N*_IFN_ = 54, *N*_Infl_ = 30, *N*_Lymp_ = 18) allowed us to raise the number of associations despite reducing the sample size (Fig. 2a). We identified 314 inflammatory-DMPs (96.18% hypomethylation), 41 lymphoid-DMPs (90.24% hypomethylation) and 659 IFN-DMPs (54.48% hypomethylation**)**, among which 214, 3, and 420, respectively, had not been previously detected when all SLE patients were pooled together (Fig. 2e). Next, we unveiled the outcomes of the enrichment analysis, emphasizing the concentration of identified genes within specific pathways. The IFN subtype showcased the most pronounced impact on genes associated with IFN pathways. Nevertheless, a persistent epigenetic IFN signature and analogous enrichment were also apparent in patients within the lymphoid and inflammatory subtypes (Fig. 2b–d and Supplementary Table 3). Additionally, synaptic functions exhibited enrichment in the set of genes with inflammatory differential DNA methylation.

We identified 71 inflammatory-dependent DMPs, 1 lymphoid-dependent DMPs and 232 IFN-dependent DMPs **(**Supplementary Fig. 1a–c and Supplementary Data**)**. For example, we observed a DNAm decrease at *SYNGAP1* and *EGR3* genes in SLE inflammatory patients (*P* < 4.08 × 10^−04^) that we did not observe in healthy controls or in patients from other subtypes (*P* < 0.05; Fig. 2f, g). For the IFN subtype, we found large DNAm differences at *IRF7* and *TRIM22* genes, (Fig. 2h, i) both of which are related to type I IFN pathways^21^.

Through EWAS interaction models, we addressed the question of whether DNAm differences at DMPs are modified by specific blood cell types. The adjustment of EWAS models by blood composition guarantees that DMPs are not explained by cell proportions, however, we did find that DNAm differences between controls and SLE patients at some DMPs is modified in relation to cell proportions, and we called this as cell-interacting DMPs. We uncovered 145 cell-interacting DMPs (*P*_*int*_ < 6 ×10^−8^), distributed as follows: 2 CD8T-interacting DMPs, 71 CD4T-interacting DMPs, 123 neutrophils-interacting DMPs, and 3 B cell-interacting DMPs. Notably, 68 of these DMPs were previously identified in our inflammatory stratified analyses, but none in the interferon or in the lymphoid groups (Supplementary Data). For example, DNAm at genes *RUSC2* and *ZNRF1* show greater DNAm differences between SLE and CTRLs in subjects with higher neutrophil and higher CD4T proportions respectively (Fig. 2k).

Intriguingly, we observed a contrast DNAm relationship with cell proportion in these DMPs, with 100% of those interacting CD4T displaying an opposite sign than those interacting with neutrophils. This could be explained by the tight negative correlation between neutrophils and CD4T cell proportions. Nevertheless, some DMPs were also found to be shared across different cell types (Fig. 2j), while 30 cell-type-interacting DMPs exhibited significance in one group but not in the other (Fig. 2j, k). We could also identify 77 novel DMPs showing cell-type-interacting DMPs. For example, DNAm at *FCGRT* gene for neutrophil, *ECE1* for B cell or *TMEM*173 for CD4T cell. Due to low number of genes, functional enrichment analysis did not yield any pathway enriched in cell-interacting DMPs (Supplementary Data).

### The relationship of autoantibody profile on the SLE epigenome

**Fig. 3:**
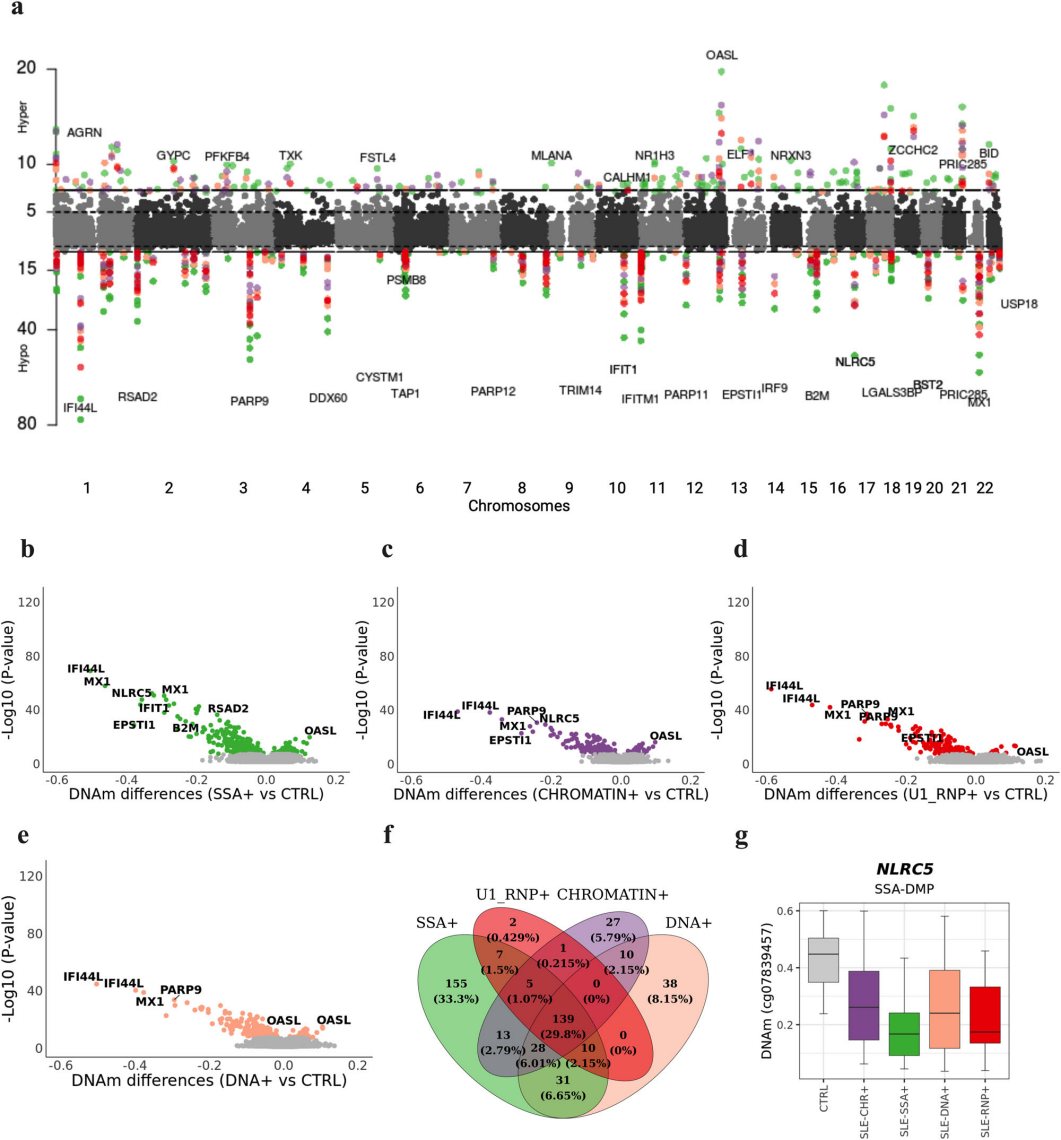
Epigenetic signatures of SLE autoantibody profiles. **a** Manhattan plot illustrating EWAS results for different groups of SLE patients according to their autoantibody profiles when compared with controls. X-axis represents the chromosomal locations of CpG sites and Y-axis represents the log10 (*P*) obtained in linear regression models. **b**–**e** Volcano Plots representing EWAS results. The X-axis represents the DNAm differences between each pair of groups tested. **f** Overlap of genome-wide significant results for each EWAS performed. **g** Example of DNAm distribution across different autoantibody groups, and healthy subjects. Colored dots represent significant DMPs after Bonferroni correction of different groups according to the legend.

We stratified patients based on their positivity for the most prevalent AAbs (*N*_SSA+_ = 54, *N*_CHR+_107, *N*_DNA_ = 61, *N*_U1-RNP_ = 40) to perform the EWAS analysis. We identified 388 anti-SSA-DMPs, 223 anti-chromatin-DMPs, 256 anti-dsDNA-DMPs and 164 anti-U1-RNP-DMPs (Fig. 3a, Supplementary Data) when compared with CTRLs, yielding a total of 466 AAb-DMPs, from which 238 had not been previously detected as SLE-DMPs, and 81 were not detected as molecular subtype-DMPs. The epigenIFNsig, and in general every CpG effect, was stronger in SSA + SLE patients for which we identified 155 DMPs not observed in the other groups (Fig. 3b–f). However, the epigenetic signature was still persistent and dominant in SLE patients positive for other autoantibodies (Fig. 3b–e). A small proportion of 27 anti-chromatin-DMPs and 38 anti-dsDNA-DMPs were exclusively observed in these groups when establishing a genome-wide significance level. However, at a significant threshold of *P* < 0.05, we could only identify one example of an anti-SSA-dependent-DMPs in the *NLCR5* gene that reached genome-wide significance level in the SSA group and was not significant in the rest. (Fig. 3g). Functional enrichment analyses on AAb-DMPs identified IFN pathways as well as antigen processing and other pathways regulatory of immune responses (Supplementary Table 4). Furthermore, the examination considered negative antibody spectra. When comparing negative AAbs with DNAm changes in healthy controls, the observed alterations were limited. The analysis predominantly highlighted IFN signals without yielding any novel discoveries (Supplementary Fig. 2a, b). Our results indicate that epigenetic signature is highly shared across SLE patients with different autoantibody profiles, and that there is little specificity on SLE autoantibody-related epigenetic signals.

### Genetic drivers of SLE epigenetic signals and mediation role of DNAm on SLE risk

**Fig. 4:**
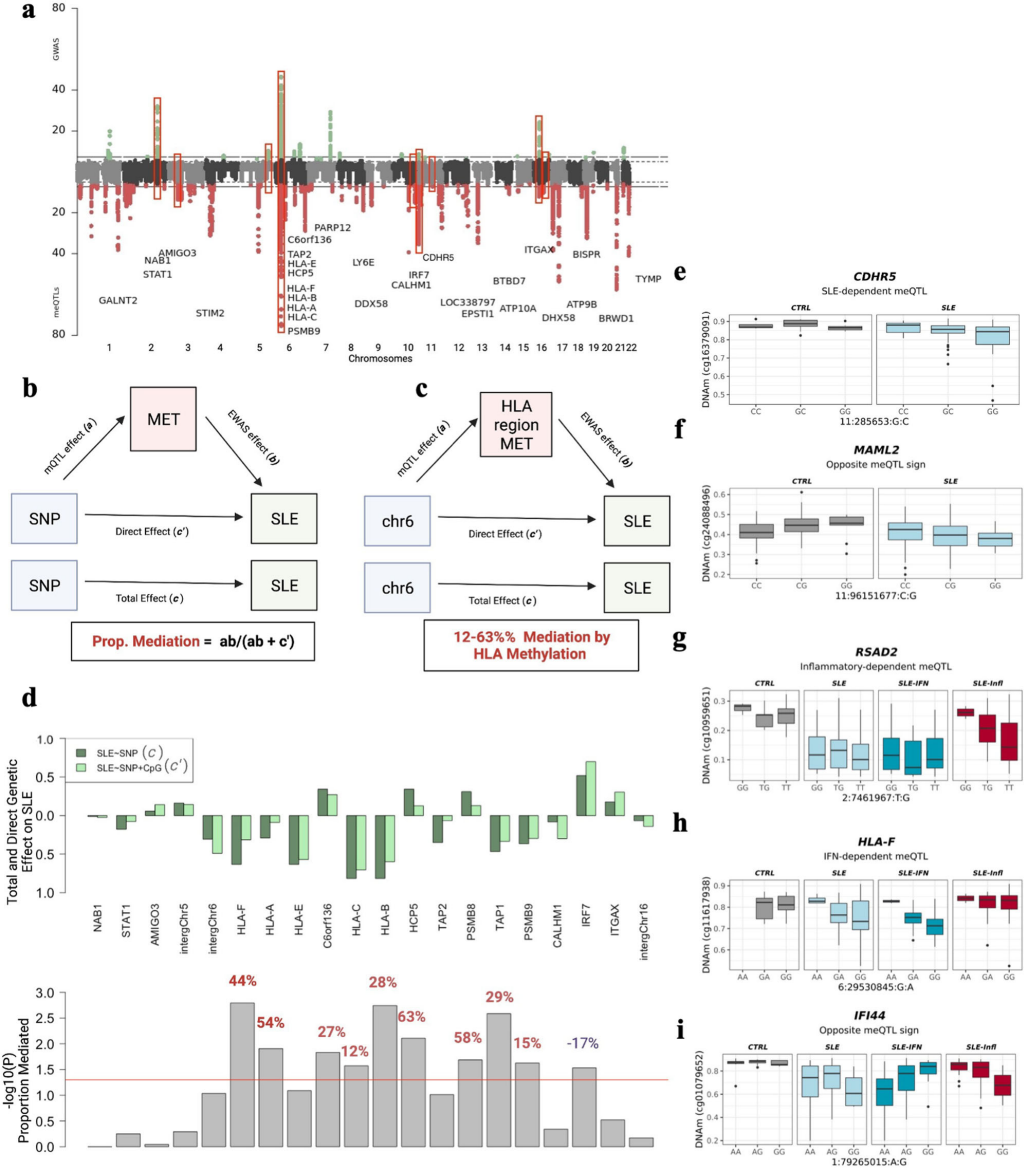
Genetic drivers of SLE-epigenetic signatures. **a** Top Manhattan plot (MP) shows GWAS results contrasting allele frequencies between a group of SLE patients (*N* = 4212) and a group of CTRL (*N* = 4065). Bottom MP illustrates meQTL results for DMPs in the whole sample. X-axis represents the chromosomal locations of CpG sites and the Y-axis represents the log10 (*P*) obtained in a logistic regression model or meQTL analyses. Genetic associations above the red line marks the statistical association at a significant threshold of *P* < 1 × 10^−06^ for logistic associations and FDR < 0.05 for meQTLs. Red boxes show overlap in GWAS and meQTL results and represent meQTLs associated with SLE diagnosis. **b** Mediation model in which SLE genetic risk is exerted partly through DNAm changes**. c** Examples of SLE-asso**c**iated SNPs in chr6 that are mediated by DNAm changes at DMPs in the HLA region. **d** Mediation results for the best SLE-meQTL-DMPs by gene. Upper barplot shows the Total and the Direct Effect of SLE-associated genetic variants. Botton barplot show the significance of the proportion mediated via DNA met resulted from mediation models. Percentage of mediation is illustrated in red below each bar only for those significant genes (*P*_proportion mediated_ < 0.05) **e**–**i** Group-dependent meQTL effects. **e** A meQTL significant effect is observed among SLE patients (FDR < 0.05) but not in the CTRL population (*P* > 0.05). **f** A meQTL significant effect is observed in SLE patients and CTRLs but with different signs. **g** A meQTL significant effect is observed among SLE from inflammatory group (FDR < 0.05) but not in the CTRL population or in the IFN group (*P* > 0.05). **h** A meQTL significant effect is observed among SLE from IFN group (FDR < 0.05) but not in the CTRL population or in the inflammatory group (*P* > 0.05). **i** A meQTL with opposite direction effects between Inflammatory-IFN subtypes.

We searched for *cis* genetic variants associated with DNAm at 148 DMPs (*cis*-meQTLs-DMPs, FDR < 0.05) (Fig. 4a). Up to 31 loci involved in *cis*-meQTL-DMPs also associate with SLE (SLE-*cis*-meQTLs-DMPs, Bonferroni significance *P* < 8 × 10^−05^; Fig. 4a**)**. Mediation analyses on SLE-*cis*-meQTLs-DMPs (Supplementary Data) revealed that SLE genetic risk is significantly reduced when DNAm is incorporated in the model (Fig. 4b), and that a significantly high proportion of SLE genetic risk at the HLA class I region (12-65% proportion mediated) is mediated by DNAm in *HLA-F, HLA-A, C6orf136, HLA-C, HLA-B, HCP5*, *TAP1* and *PSMB9* genes (Fig. 4c, d**)**. Other SLE-associated SNPs such as those located in *STAT1* or the intergenic region at chromosome 5 nearby microRNA mir-146 also showed a significant indirect effect of SNP on SLE suggesting a mediation role of DNAm, but the proportion mediated did not reach statistical significance (*P* > 0.05), probably due to the low sample size. Interestingly, for SLE-associated genes such as *IRF7* and *ITGAX*, the SLE genetic effect increased when DNAm was included in the model, suggesting a cofounded or more complex relationship between DNAm, genetic variation and SLE (Fig. 4d).

### Context-dependent meQTL regulatory function in SLE

We discovered *cis*-genetic variants associated with DNAm with dependency on disease status or molecular subtype by the identification of meQTL in different groups together with significant interaction effects. We identified SLE-dependent meQTLs for 394 DMPs among which significant disease-dependent effects were observed in *CDHR5* and *MAML2* (Fig. 4e, f). Likewise, we discovered inflammatory- and IFN-dependent meQTLs for 283 and 316 DMPs, respectively (Supplementary Data). For instance, we observed genetic regulation on DNAm in *RSAD2* gene in inflammatory SLE patients, but not in the IFN group or in controls where *RSAD2*-DNAm does not show a relationship with the genotype (Fig. 4g). IFN-dependent meQTL effects were observed for example for *HLA-F* (Fig. 4h). Intriguingly, we also identified meQTLs with strong opposite effects (op-meQTLs) between SLE and CTRLs (119 DMPs) or between the IFN and inflammatory subtypes (35 DMPs) among which *IFI44* gene shows the greatest opposite effect (Fig. 4i). Some genetic variants involved in context-dependent meQTLs were associated with SLE at a Bonferroni-corrected threshold (*P* < 1.2 × 10^−04^) (Supplementary Data). The strongest SLE-associated SNPs involved SLE-dependent meQTL that associated with DNAm at *HLA-B* and *HLA-E* genes. Interestingly, we also identified strong genetic-disease interaction (Pint = 6.5×10^−05^) in the regulation of DNAm within the *CDHR5* gene at chromosome 11, that involve SNPs strongly associated with SLE (*P* = 1.9 × 10^−07^; Fig. 4e).

### SLE-associated epigenetic signals correlate with transcription factor activity and cytokine production

**Fig. 5:**
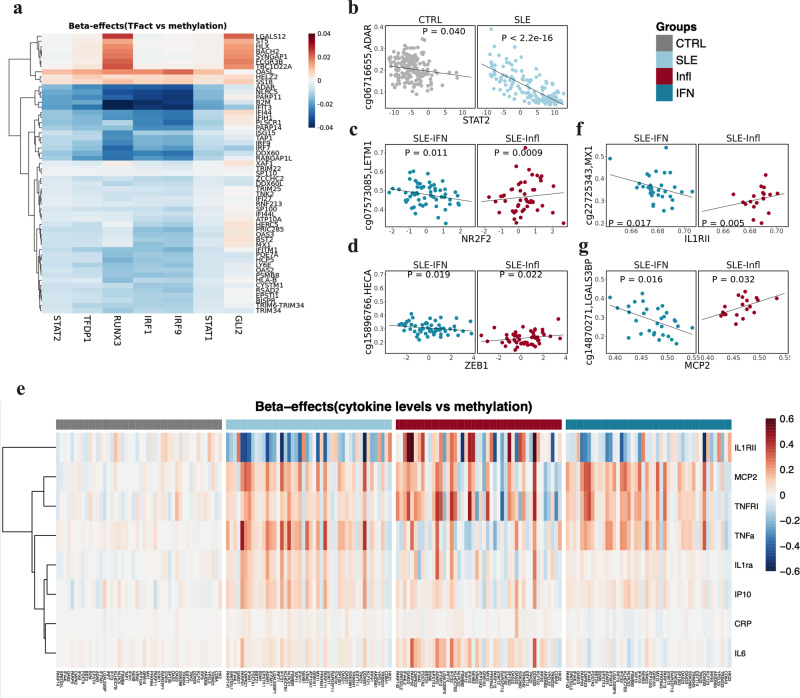
Relationship between SLE-epigenetic signatures, transcription factor activity, and cytokine production. **a** Heatmap representing SLE-dependent associations between DNAm at DMPs and TFact inferred from RNAseq data. **b** SLE dependent example showing the effect between TFact STAT2 and DNAm at *ADAR* gene vs CTRL. **c**, **d** Subtypes dependent examples showing the effect between TFact ZEB1 and NR2F2 and DNAm at *SPATS2L* and *LETM1* genes. **e** Heatmap showing the effect distribution of CpGs-genes associated to cytokine levels exhibiting group specificity. Color gradient from blue to red correspond to effect sizes. **f**, **g** Examples for cytokine opposite associations at DNAm levels for inflammatory and IFN subtypes.

Transcription factor (TF) binding has an important role in shaping DNAm levels and vice versa^22^. Here, we identified up to 61 different TFs whose activity correlated with DNAm at several DMPs (*P* < 0.05) (Supplementary Data). Interestingly, the large interaction between DNAm and TF activity (TFact) for *IRF9*, *IRF1*, *STAT2*, *STAT1*, *STAT3*, *TFDP1*, *FOXM1*, *E2F3*, *E2F2*, *GLI2,* and *RUNX3* was restricted to SLE patients (Fig. 5a, b). While, TFs of IRF and STAT family have a well described role in the activation of IFN pathways and are associated with DNAm at IRGs. The TFact of TFDP1, E2F3, and FOXM1 correlated also with DNAm at IRGs such as *IFI44L, MX1, TRIM22*, and *ISG15*. Interestingly, *RUNX3* activity was associated with DNAm at genes such as *FCGR3B, HLX, LGALS12, BACH*, and *SYNGAP* which are not IFN-regulated. Intriguingly, we observed differential DNAm-TFact associations when comparing between inflammatory and IFN SLE patients. For example, NR2F2 TFact is strongly negatively associated with DNAm at the *LETM1* gene, but such effect is not observed in the IFN subtype (Fig. 5c). Likewise, ZEB1 TFact is associated with DNAm at *LGALS9, RSAD2*, *TMEM123, HECA,* and *IFI44L* genes only in IFN patients (Fig. 5d).

We explored whether DMPs could be associated with cytokine production in each molecular group. In total, 82 DMPs were significantly associated with levels of 8 cytokines (*P* < 5 × 10^−05^). The strongest (and negative) associations were found between IL1RII levels and SLE-DMPs at a number of IRGs such as *PARP9* and *IFI44L*. We found SLE CpG-cytokine association for TNFa (*VRK2*, *PARP9*), IL1ra (*BST2*, *ATP10A*), IL1RII (*IFITM1*, *ARID5B*), MCP2 (*IFI44L*, *CMPK2*), and IP10 (*NLRC5*, *B2M*) which were not observed in CTRLs. We also observed inflammatory-dependent association for TNFRI (*RAPGEF1*) and CRP (*OAS3*) (Supplementary Data). Figure 5e illustrates the strength of the group-dependent associations. It can be observed that associations differed between groups and that for some CpGs, for cytokines such as IL1RII, these were stronger within the inflammatory as compared to the IFN subtype and stronger than CTRLs. For example, IL1RII was negatively associated with DNAm at *MX1* in IFN subtype but it is positively associated in the inflammatory subtype (Fig. 5f), similarly MCP2 showed opposite effects at the DNAm level for the *LGALS3BP* gene for the inflammatory and IFN subtypes (Fig. 5g). Altogether, our results indicate that the relationship between DNAm, TFact and cytokine production is determined in a subtype-dependent manner.

### SLE-associated epigenetic signals inform drug discovery

**Fig. 6:**
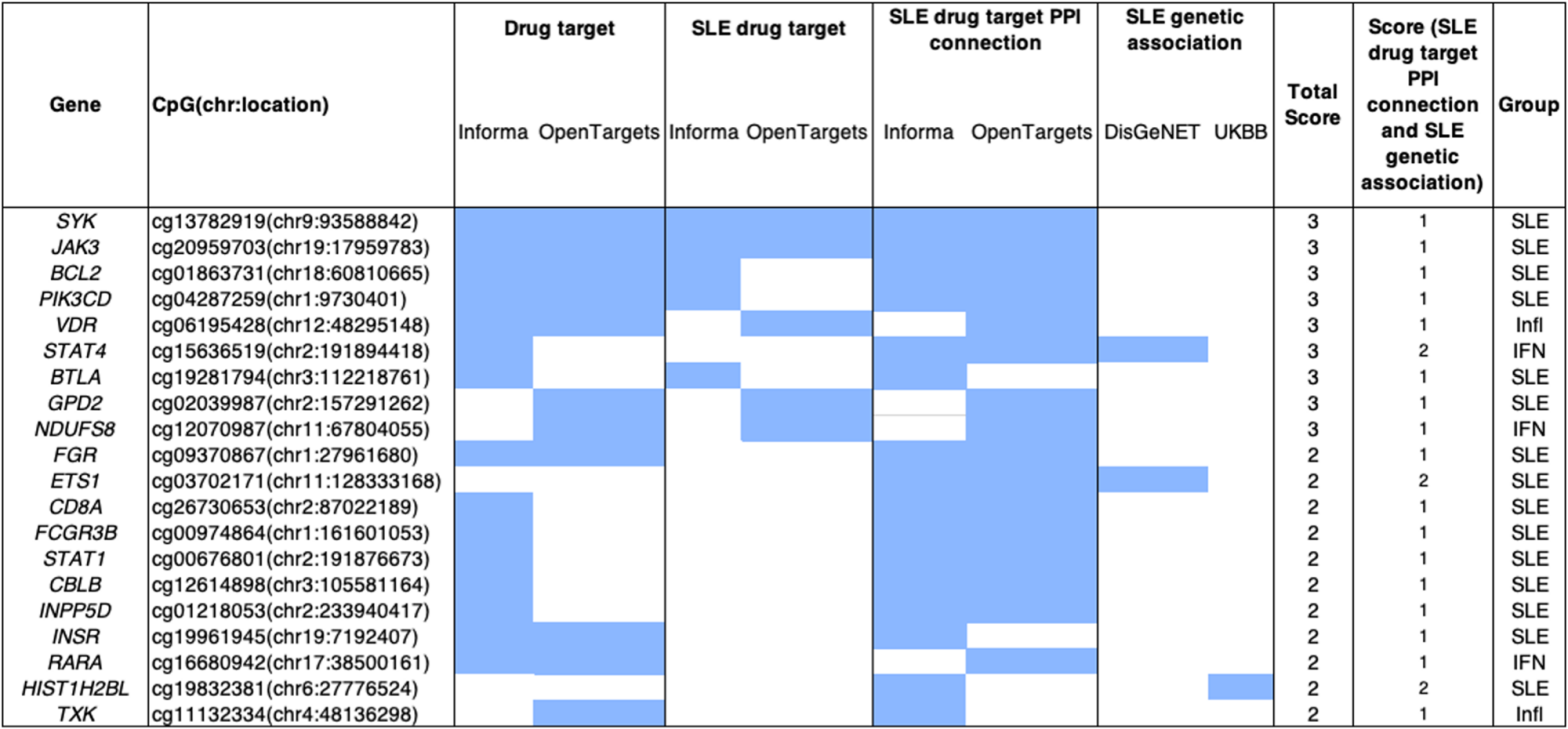
Representative SLE Epigenomic signature genes. We list the top 20 (of the 549 genes from the identified 974 CpG sites) based on summary of gene total scores derived from individual criteria (filled box indicates criterion satisfied). Filled boxes indicate an overlap with the data source described in each column. For full results, see Supplementary Data.

We observed an enrichment of the list of unique 549 SLE differentially methylated genes (DMG) among known drug targets (Fold enrichment of 1.4, *P* < 0.01 in phase 1 or above in OpenTargets and Informa databases), and identified a total of 62 DMG being known drug targets, including 8 known SLE drug targets: *SYK*, *JAK3*, *BCL2*, *PIK3CD*, *VDR*, *BTLA*, *FGR*, *GDP2*, and *NDUFS8* (Supplementary Table 5 and Fig. 6).

To identify and prioritize candidate novel SLE gene targets, we collected different features of each gene and ranked them based on two criteria: (i) previous evidence of genetic association to SLE (ii) “network proximity” (Fig. 6, see “Methods” section for scoring details). We showed strong enrichment of validated targets within the groups of genes with scores equal or higher than one (Supplementary Fig. 3). Using the different annotations available for each gene of the list, we could prioritize genes of interest and focus on potentially novel targets (see **discussion**) for SLE, and for different SLE subtypes. For example, we observed that 4 of the known drug targets (*VDR*, *ALOX5AP*, *ITGA5*, and *ECE1*) overlapped with genes that are differentially methylated only in the inflammatory SLE patients. Likewise, 17 genes (*CLU*, *CETP*, *TSHR*, *ITGA2*, *ACACA*, *TNK2*, *STAT4*, *PSMB8*, *SCN8A*, *INPP5D*, *BMPR1A*, *TAP1*, *TYMP*, *QPCT*, *GPD2*, *PSMB9,* and *LAMB1*) overlapped with interferon specific epigenetic signatures.

## Discussion

In this study, we present the first integrative EWAS that contrasts genome-wide DNAm data in several stratifications of SLE patients and integrates results with genetic, transcription, clinical, and serological data.

Stratifying SLE patients across homogenous molecular subtypes allowed us to significantly increase statistical power expanding the epigenetic signals and reporting new loci associated with SLE that had not been revealed when analyzing SLE heterogeneous populations. The largest effects were mostly found at interferon-related genes as for example *IFI44L*, *MX1*, *NLRC5*, *PARP9*/*DTX3L*, which has been extensively identified in previous SLE-EWAS across most blood cell types and in other SADs^7^. Here we show that the epigenetic IFN signature is present across all SLE molecular subtypes at different intensities, however we could discover some epigenetic signals exclusively in those SLE patients from the IFN subtype, as DMPs at *IRF7* and *TRIM22* genes. *IRF7* has been established as an SLE genetic risk locus that alters IFN type I expression^21^ and TRIM molecules have been studied as autoantigens in some autoimmune diseases, especially in Sjögren’s syndrome (SS) where *TRIM22* protein showed little or no immunoreactivity in a sub-population of SS patients^23^ suggesting that *TRIM22* was acting as a potential SSA60 regulated gene. Importantly, we also discovered epigenetic signals that are specific of SLE patients with an inflammatory molecular profile, as those in the *EGR3* and *SYNGAP* genes. *EGR3* is a member of a zinc finger transcription factor that plays an important role in regulating immune responses inducing also the expression of anti-inflammatory cytokines such as IL-10 and TGFB1^24^, however the role of *EGR3* in autoimmunity it is not clear yet. *SYNGAP1* encodes a Ras GTPase activating protein that is member of the N-methyl-D-aspartate receptor complex and has been identified as a differentially methylated gene associated to gene expression in systemic sclerosis (SSc)^25^.

By incorporating interaction terms in the EWAS model, we could identify DMP which DNAm differences between SLE and controls is modified by specific cell type proportions, most of them implied neutrophil-interacting DMPs, and implied CpGs discovered in the SLE inflammatory cluster. Our findings are in agreement with previous research by *Barturen* et al. ^8^, which demonstrated specific neutrophil epigenetic signature associated with the inflammatory molecular subtype. On the other hand, the fact that cell-interacting DMS were not that spread among IFN-related CpGs do suggest that these changes are shared in the same magnitude among different blood cell type, as it has been described before.

We also observed a noteworthy opposite effect in cell-interacting DMPs between neutrophils and CD4T cells. This contrasting methylation pattern aligns with the known regulatory role of neutrophils in restricting CD4 + T cell expansion during adaptive immune responses^26^. We also identified unique DMPs that are only observed in one particular cell type. For instance, we noted a CD4T-interacting DMP within the *ZNRF1* gene, encoding an E3 ubiquitin-protein ligase involved in neural-cell differentiation, with known associations to diabetic retinopathy^27^. Additionally, genes such as *RUSC2* and *RBL1*, contributing to the interaction with neutrophils, have previously been linked to lupus nephritis^28,29^. These cell-interacting DMPs results shed light on the role of cell type differential DNAm in immune-related disease processes and demonstrated that new SLE-associated epigenetic signals can be revealed. Further analyses based on cell-type derived DNAm in larger sample sizes will expand these results.

Our stratification approach also included an exploration of the AAb relationship on the SLE epigenome. The DMPs with largest effects were found within SSA positive SLE patients. Our previous work showed that the epigenIFNsig in Sjogren’s syndrome is restricted to SSA positive patients and driven by HLA genetic variation^9^. However, in SLE many of the signals observed are shared across SLE patients with different AAb profiles, which suggests that in SLE, the epigenetic signature is not AAb-specific or AAb specificities are highly correlated.

One of the most important findings of this work is that SLE-associated genetic variation might exhibit its risk through DNAm changes, this is especially true for SLE-associated SNPs within the HLA region. Our findings strongly suggest that beyond the impact on antigen presentation, genetic risk at the HLA region is also mediated through the epigenetic and transcriptional alterations of many genes residing within the HLA such as *HLA-F, HLA-A, HLA-C, HCP5, PSMB8, TAP1*, and *PSMB9*. These results are in concordance with previous work showing an overexpression of these genes in SLE patients^7^. Building upon these findings another study found that genetic variation in the HLA region can influence the transcriptional regulation of genes in memory CD4 + T cells during activation^30^. Outside the HLA region, our mediation results also support an indirect effect of SLE-associated genetic variation via DNAm within *STAT1* and *microRNA146* gene, previously genetically associated with SLE. However, given the small effects of these SNPs on SLE, and the sample size analyzed here, confirmatory studies are needed to provide conclusive results, and larger sample sizes are needed to explore differential mediation effects between different SLE molecular subgroups. In the same line, analyzing public repositories of genetic associations by means of Mendelian Randomization approaches would help to resolve puzzling relationships as those observed for genetic variation, DNAm and SLE risk at the *IRF7* gene.

Our findings show that there exists a clear influence of disease and molecular status on the genetic architecture of DNAm, given the fact that DNAm at a large proportion of DMPs is regulated by meQTLs but exclusively in the SLE population context or in certain molecular states. For example, this is the case for *CDHR5* gene which DNAm is regulated by genetics only in the IFN and SLE subtypes, respectively. Interestingly, our results show that genetic variants involved in *CDHR5* meQTLs are also associated with SLE risk. The SLE-associated SNPs represent novel disease variants as they have not been previously identified in GWAS and are likely to mediate their effects trough DNAm changes. Previous studies have identified *CDHR5-*SNPs located close to *IRF7*^31,32^ and associated with systemic sclerosis (SS)^32^. The candidate gene *CDHR5* is a member of the cadherin family which interacts with the β-catenin pathway^33^. These observations are in agreement with a growing body of evidence that highlights that genetic effects are largely context and time-specific, and implicates that future research and data collection of pathological and molecular status within longitudinal larger autoimmune populations will be able to decipher many more genetic variants with important regulatory functions in autoimmunity^34^.

Transcription factor activity has been linked to SLE recently^24^. In this work, we identified a group of TFs whose activity was associated with SLE-associated DNAm changes. The activity of STAT and IRFs regulators show the strongest association with DMPs at IRGs, and here we show that this relationship is specific of SLE population and not observed in healthy individuals. We also reveal other TFs not previously implicated in SLE, such as TFDP1, E2F3, and FOXM1 whose activity associates with DNAm at IRGs. On the contrary, we identified that *RUNX3* activity, a susceptibility gene in SLE and systemic sclerosis (SSc)^35,36^, correlates with DNAm at non-IFN-related genes. Our results suggest that TFact could play a relevant role in autoimmunity by altering epigenetic programs.

In SLE pathogenesis, an inflammatory cascade is mediated by altered cytokine production. Despite the growing recognition of the high potential of DNAm changes as surrogate and biomarkers of pro-inflammatory proteins, especially in ageing phenotypes^37,38^, no study, to the best of our knowledge, had reported epigenetic-cytokine correlations within autoimmune diseases. Here we show a strong association in the SLE population between inflammatory cytokines and methylation changes, not observed within healthy individuals, being IL2RA and IP10 the cytokines showing the strongest association with the epigenIFNsig. Our results also show that many epigenetic-cytokine associations are only observed in certain molecular contexts.

Finally, we discovered several interesting potential new drug targets based on the SLE epigenetic profiles. Among the novel SLE candidate genes with higher score is *STAT4*, which is in direct PPI connections with SLE drug targets and exhibit strong genetic associations^39–44^. Another interesting target candidate is the transcription factor *ETS1*, which also had a significant strong SLE genetic association^45^ and which is in direct PP interaction with SLE drug targets such as *JAK3*. This protein has been observed to suppress T follicular helper type 2 cell differentiation and halt SLE onset^46,47^. Of interest, *FGR*, a member of the Src family of protein tyrosine kinases (PTKs) was scoring high for being a drug target for non-SLE conditions, but in close PPI connections with SLE validated targets, making it a potential candidate for drug repositioning. It functions as a positive regulator of cell migration and regulates cytoskeleton reorganization via *RAC1* activation. It also phosphorylates *SYK* and promotes *SYK*-dependent activation of *AKT1* and MAP kinase signaling. *SYK* is one of the top genes in the candidate drug target gene list for SLE^24,48^, as *STAT4* is for the IFN subtype and *VDR* for the inflammatory.

This study has some limitations. First, our study focused on discerning molecular alterations within samples derived from whole blood. This composite includes a variety of immune cell types, many of which play direct roles in the autoimmune processes associated with SLE. The statistical methodology employed in this work allowed us to report epigenetic changes present with and without dependency on cell type abundance. However, it is crucial to note that our investigation did not unveil specific molecular changes exclusive to particular cell types and the identification of specific pathways influenced by these cell type-dependent epigenetic changes remains elusive due to the limited number of significant DMPs found in this analysis. Despite this limitation, exploring these potential pathways could be a compelling avenue for future research endeavors, particularly with larger sample sizes and DNAm data derived from sorted cells or single cell analyses. We were also unable to detect unique epigenetic alterations linked to specific AAbs positivity. For most SLE patients, AAbs are not mutually exclusive, implying that individuals positive for one AAb are likely also exhibiting other AAbs. Future investigations focusing on AAbs combinations in autoimmune diseases will enhance our comprehension of the epigenetic variations associated with AAb profiles and SLE.

To wrap up, our study disentangles epigenetic signatures in SLE with regards to different heterogeneity aspects and identifies potential novel drug targets. By means of integrative multi-omics stratifying analyses we show that the strong association between epigenetics, genetics, TFact and cytokine production is highly dependent on disease and molecular context. Our results serve to motivate future epigenome-wide and genomic studies to molecularly stratified autoimmune patients in order to gain biological and statistical power and unravel novel genes and molecular mechanisms involved in disease pathogenesis.

## Methods

### Samples

We included 292 SLE patients and 320 healthy controls (CTRL). The sample was divided into a discovery set (213 SLE patients and 221 healthy controls with DNAm data based on the EPIC array), and a replication set (79 SLE patients and 99 with DNAm data based on the 450 K array) from the PRECISESADS project^8^. Supplementary Table 1 describes the main characteristics of the study sample, together with the groups and traits analyzed. Blood cell proportions were obtained using flow cytometry^49^ while autoantibodies, cytokines and other inflammatory mediators were analyzed from serum. Autoantibodies (anti-chromatin, anti-dsDNA, anti-U1RNP, anti-SSA/Ro, anti-SSB/La, anti-SM, anti-β2 glycoprotein 1, anti-β2 microglobulin, IgG anti-cardiolipin, IgM anti-cardiolipin, rheumatoid factor, and anti-ENA), cytokines and inflammatory mediators (BAFF, BLC, CRP, FASL, GDF15, IL1RII, IL1RA, IL6 IP10, MCP2, MCP4, MIP1B, MMP2, MMP8, TARC, TGFβ1, TNFRI, and TNFα) were measured in serum samples as described by Barturen et al.^8^.

### Ethical declaration

An ethical protocol was prepared, achieving consensus among all partners, both academic and industrial, and was subsequently translated into the languages of all participants. It received approval from each local ethical committee at the clinical recruitment centers, as did all experimental protocols. For a comprehensive list of local committees and centers involve in PRECISESADS, please refer to Supplementary Note 1. All recruited patients were 18 years of age of older and provided signed informed consent forms. All methodologies were conducted in strict accordance with relevant guidelines and regulations, adhering to the standards set forth by the International Conference on Harmonization and Good Clinical Practice, as well as ethical principles stemming from the Declaration of Helsinki (2013). Confidentiality of records containing identifiable information was upheld in accordance with EU Directive 2001/20/EC and the applicable national and international data protection requirements in each participating country.

### DNAm profiling

DNAm data was produced using the Illumina Infinium HumanMethylationEPIC BeadChip array and the Illumina Infinium HumanMethylation450 BeadChip array, which covers up to 850,000 and 485,512 CpG sites respectively. DNA was extracted from peripheral blood samples from which the genome was amplified and hybridized to the Illumina arrays. Standard methodological procedures for quality control and probe filtering were performed as described previously^50^. Samples were excluded based on the detection P criteria > 99%, poor bisulfite conversion based on control dashboard check, and sex mismatches according to failed chromosome X and Y clustering. Probes were filtered out based on detection *P* > 0.01 in > 95% of samples. Additionally, probes located at the X and Y chromosomes were separated in different datasets to avoid gender bias. Probes with genetic variants at their CpG sites were also excluded. After applying these filtering steps we obtained 776,284 and 433,337 autosomic probes in the discovery dataset and in the replication dataset, respectively. After QC, the raw methylation beta values were background corrected and normalized using the functional normalization within the meffil R-package. A beta value ranging from 0 to 1 was used to measure DNAm in whole blood, being 1 the methylated status with 100% of cells being methylated at a given CpG and 0 the unmethylated status with 0% of cells being methylated.

### Genetic profiling

Genotyping was performed using InfiniumCore from Illumina. Imputation was performed using the Michigan Imputation Server^51^ and Haplotype Reference Consortium as reference panel^52^. Filtering of genetic variants and quality controls were performed by PLINK^53^. A total of 4,553,097 variants with a minor allele frequency (MAF) higher than 0.05 were used for subsequent analyses.

### Epigenome-wide association analyses

We performed a series of EWAS interrogating DNAm differences between groups at each autosomic CpG site. We used linear regression models adjusting by sex, age and blood cell composition (B cells, CD4 T cells, CD8 T cells, monocytes, neutrophils and natural killer cells) as well as technical confounder effects (Sample_Plate and Sample_Position). SLE-associated differentially methylated CpG positions (SLE-DMPs were identified comparing DNAm between SLE patients and CTRLs. We stratified SLE patients into the molecular subtypes described in *Barturen* et al.^8^ and by autoantibody (AAb) profile and compared their DNAm separately with CTRL. In total, 30 SLE patients were classified into the inflammatory subtype, 18 into the lymphoid subtype and 54 to the IFN subtype in the discovery cohort (Fig. 1) and 11, 7, and 19 SLE patients in the replication cohort as previously defined by Barturen et al. ^8^. We only performed EWAS on those AAbs that exhibited positivity in more than 20 SLE patients and corrected models for the presence of all other autoantibodies. We applied a Bonferroni significance threshold (*P* < 5 × 10^−08^) to claim genome-wide significance. We assessed replication (*P* < 0.05) in an independent cohort from based on 450K-methylation data and determined replication rate for those CpGs present in both arrays. To determine group-dependent differentially methylated positions (DMPs) (ex. SLE-dependent DMPs), we used strict significant threshold for the specific DMP passing a Bonferroni threshold in one group while exhibiting a *P* > 0.001 in the others. We separately identify cell-type-interacting DMPs through linear regressions models with an interaction term between cell proportion and disease or molecular status, while adjusting by the same covariates used in the EWAS model.

### Functional enrichment analyses

Gene-set enrichment function analyses were performed to reveal if genes annotated to DMP were enriched in particular functional pathways. We used the Reactome database^54^ implemented by *enrichPathway function* in R library ReactomePA^55^. As background distribution, we used all genes interrogated in the EPIC array. We separately analyzed genes showing DMP with hypomethylation and hypermethylation events.

### Methylation quantitative trait *loci* analysis

We performed methylation quantitative trait locus (meQTL) mapping using the Matrix eQTL R package^56^, by means of linear regressions in which the minor allele dosage effect on DNAm was tested while adjusting for age, sex, batch effects, cells proportions, the first genetic principal component, and disease status. We defined *cis*-meQTLs as single nucleotide polymorphisms (SNPs) located no farther than 1 Mb from the interrogated DMP. *Cis*-meQTLs were separately discovered in different groups: i) whole sample, ii) SLE patients, iii) healthy controls, iv) inflammatory SLE patients, and vi) IFN SLE patients. Lymphoid subtype was discarded from this analysis due to the low sample size. We used a permutation-based False Discovery Rate (FDR) < 0.05 to claim significance. We investigated the interaction effect between SNP and group for the following groups: SLE vs CTRL and inflammatory vs IFN. To determine group-dependent meQTLs we used the following statistical conditions: FDR < 0.05 in one group and *P* > 0.05 in the other group and *P* < 0.05 significant interaction effect. We also looked for meQTLs with an opposite and significant genetic effect in different groups, with the ensuing conditions i) FDR < 0.05 in one group AND *P* < 0.05 in another group, or ii) *P*_interaction_ < 0.05 AND *P* < 0.05 in both groups AND FDR _interaction_ < 0.005.

### Genetic association and mediation analyses

We ran a genome-wide association study (GWAS) in an European sample of 4212 SLE patients and 4065 controls previously described^57^, and extracted the results for those SNPs involved in meQTLs regulating 148 DMPs. We established a Bonferroni significance threshold of *P* < 0.05/148 = 0.00033 to claim statistical significance. We performed mediation analyses with the R package mediation using the method development by Imai et al. ^58^ to quantify mediation effect of DNAm on SLE genetic risk. We report the Total Genetic Effect of the SNP on SLE (**c**), the Direct Genetic Effect of the SNP on SLE from a model adjusted by DNAm (**c’**), and estimated the proportion of the Total Genetic Effect on SLE explained by DNAm Mediation by the formula: *Prop. Mediated* = (a*b)/(a*b + *c’)*, where ***a*** is the effect of the SNP on DNAm and ***b*** is the effect of DNAm in SLE. Test statistics for these measurements were estimated by 10,000 Monte Carlo simulations.

### Epigenetic associations with cytokine levels and transcription factor activities (TFact)

We inferred TFact for 119 transcription factors in the PRECISESADs data using whole blood RNAseq data and the R package DoRothEA^59^ as previously described^24^. Linear regression models were performed between DNAm at DMP and log transformed cytokine levels for 18 different inflammatory cytokines (see supplementary Table 1) or with TFact in different groups (SLE, CTRL, inflammatory, and IFN) and corrected for the same covariates used for the EWAS/meQTLs. A Bonferroni threshold of *P* < 0.05/1198 = 4E-5 was used to claim statistical significance.

### Identification of drug targets within epigenetic signals

We used different informatics platforms and data sources (Supplementary Methods) to identify drug targets within epigenetic signals. Specifically, for each gene in the list of the 549 differentially methylated genes, a “Total Score” was calculated as the sum of individual sub-scores, as described below (see also Fig. 6):

(I) “known drug target” in OpenTargets OR Informa (clinical phase 1 or above), sub-score = 1;
(II) “known drug target for SLE” in OpenTargets OR Informa (clinical phase 1 or above), sub-score = 1
(III) having direct interactions with an SLE drug target (from OpenTargets OR Informa) in the PPI network, sub-score = 1
(IV) reported in DisGeNet to have GDA score of association with SLE > 0.3, sub-score = 1
(V) reported from UK BioBank with significant genetic variant associations (*P* < 5 × 10^−08^) to SLE, sub-score = 1

### Reporting summary

Further information on research design is available in the Nature Research Reporting Summary linked to this article.

### Supplementary information

Supplementary Material

Supplementary Data

Reporting Summary

## Data Availability

ELIXIR Luxembourg (https://elixir-luxembourg.org) serves as the host for the data. Due to restrictions mandated by the ethics committee and/or informed consent agreements with participant, we are unable to deposit the data into a secure access-controlled repository. Upon request, the data is accessible, and the access procedure is outlined on the data landing page with the following 10.17881/th9v-xt85. Online availability of DNA methylation data can be found at https://bioinfo.genyo.es/precisesadsdata/.

## Code availability

All code utilized for data analysis and figure generation will be made accessible upon request. The computational tools are built within the R framework and detailed in the Methods section. Interested parties can contact the corresponding authors for access to the code. We used the latest R (R version 4.3.1) and associated package versions: *ReactomePA* (version 1.44.0), *MatrixEQTL* (version 2.3), *DoRothEA* (version 1.14.1) and *ggplot* (version 3.4.3) and *karyoploteR* (version 1.28.0) for figures.
