# Supplementary material for: Molecular subtypes explain lupus epigenomic heterogeneity unveiling new regulatory genetic risk variants
Source: NPJ Genom Med. 2024 Jul 16;9:38. doi: 10.1038/s41525-024-00420-0 (PMC11252280; doi:10.1038/s41525-024-00420-0)
Supplement: Supplementary file 1 — Supplementary Material [file 41525_2024_420_MOESM1_ESM.pdf]

Molecular subtypes explain lupus epigenomic heterogeneity unveiling new regulatory genetic risk variants

Supplementary Material

Supplementary Figures

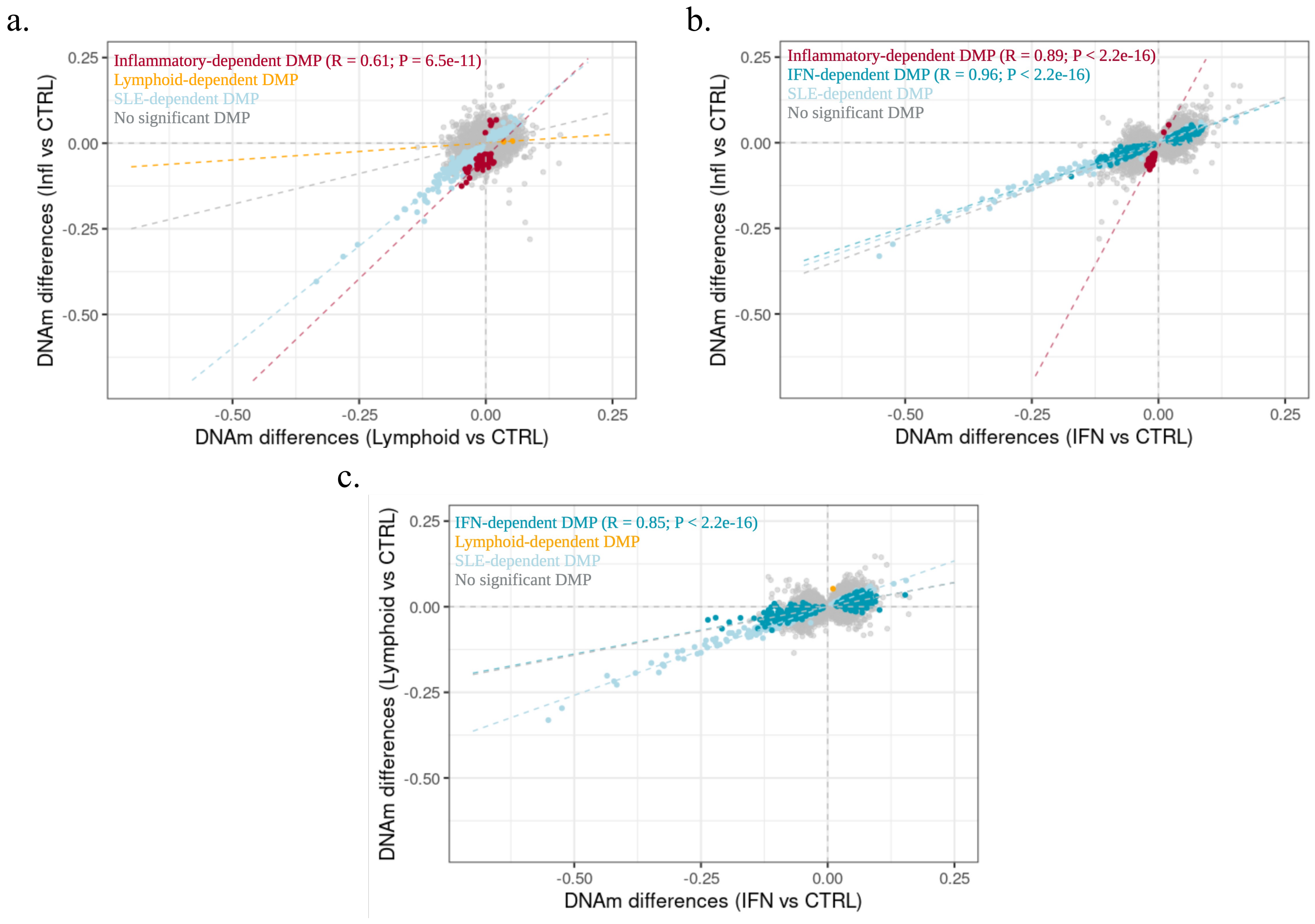

Supplementary Figure 1. Effect Size Comparison comparing results from different EWAS. **a**. Effect size comparison between inflammatory-dependent DMP and lymphoid-dependent DMP. **b**. Effect size comparison between inflammatory-dependent DMP and IFN-dependent DMP. **c**. Effect size comparison between IFN-dependent DMP and lymphoid-dependent DMP.

a.

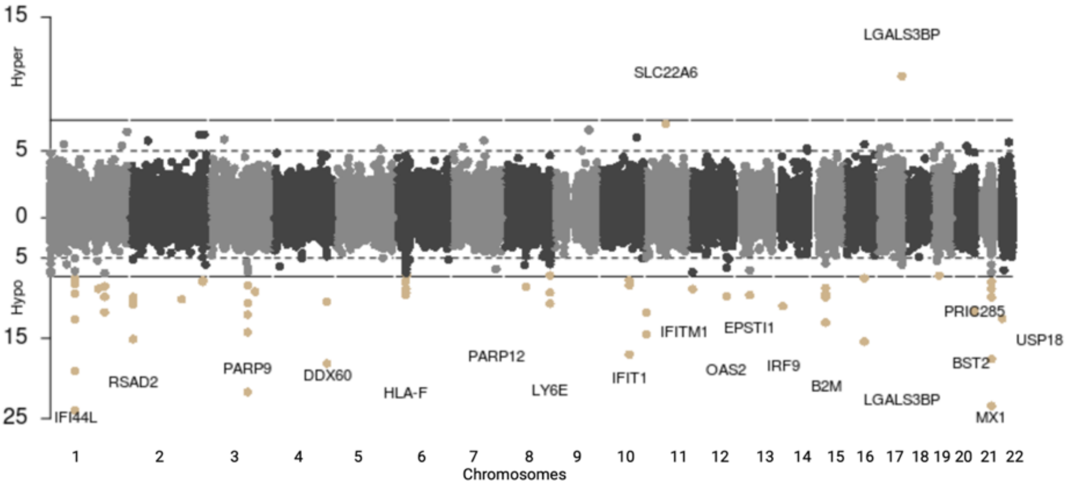

b.

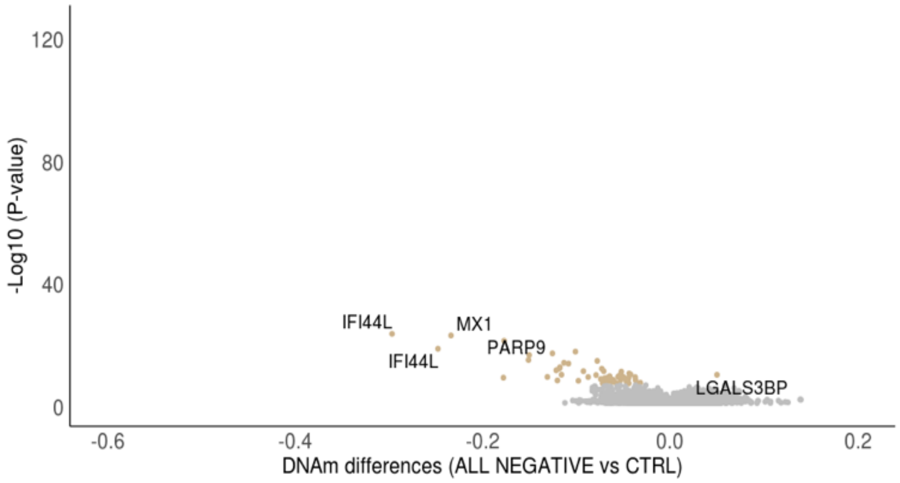

Supplementary Figure 2. Epigenetic signatures of SLE negative autoantibody profiles. **a**. Manhattan plot illustrating EWAS results for negative autoantibody SLE patients compared with controls. X-axis represents the chromosomic locations of CpG sites and Y-axis represents the log10 (P) obtained in linear regression models. **b**. Volcano Plots representing EWAS results. The X-axis represents the DNAm differences between each pair of groups tested.

a.

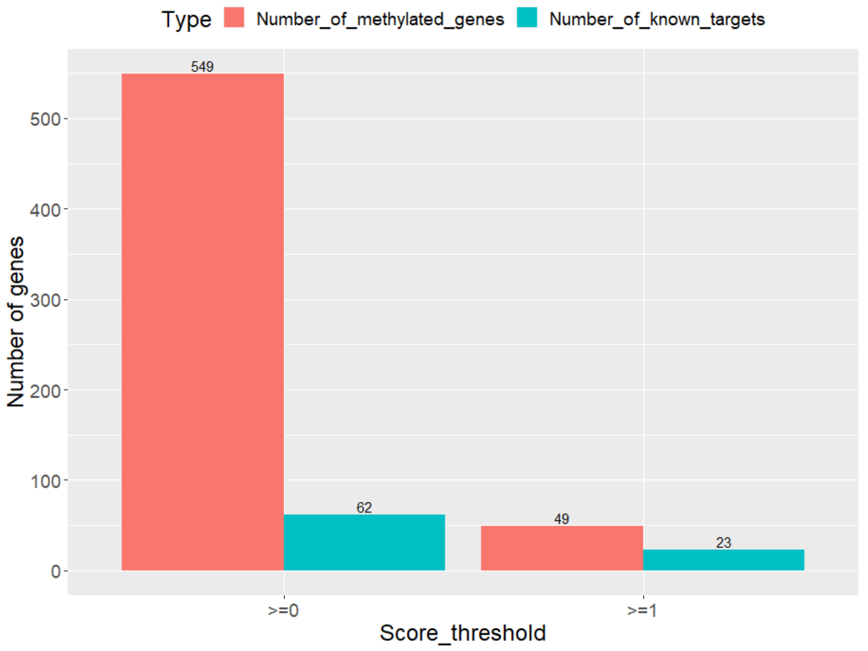

b.

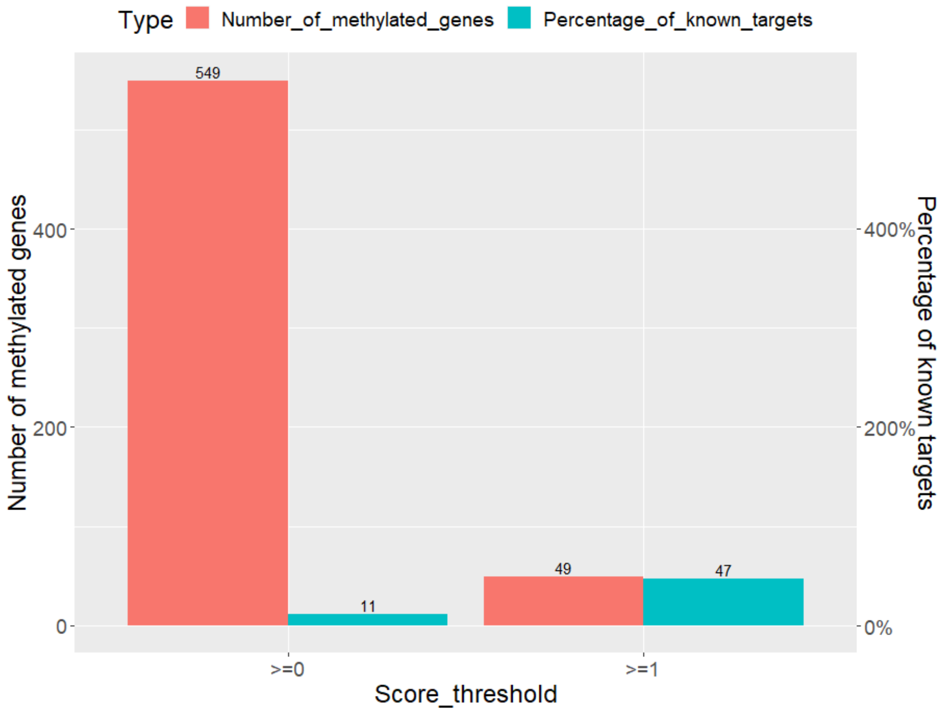

Supplementary Figure 3. Relationship between SLE Epigenomic signature genes and known drug targets. We set different score thresholds to reduce the number of SLE methylated genes and compare them with known drug targets. The score threshold was set based on the portion of total scores that derived from PPI direct interactions with an SLE drug target and SLE genetics association analysis (Figure 6). **a.** Bar plot with number of methylated genes and known drug targets that are in these

methylated genes under different score thresholds. **b.** Bar plot with number of methylated genes and the rate of known drug targets in these methylated genes under different score thresholds.

Supplementary Table 1. Descriptive table

		N EPIC		N 450K	
		**SLE**	**CTRL**	**SLE**	**CTRL**
	**All individuals**	213	221	79	99
	**Inflammatory cluster**	30	_	_	_
	**Lymphoid cluster**	18	_	_	_
	**Interferon cluster**	54	_	_	_
**AUTOANTIBODIES**	**SSA+**	54	4	19	1
	**SSA-**	133	211	39	70
	**SSB+**	16	0	2	1
	**SSB-**	172	215	56	70
	**B2G+**	24	1	4	0
	**B2G-**	177	216	54	71
	**B2M+**	16	6	4	1
	**B2M-**	185	211	54	70
	**CHROMATIN+**	107	4	29	2
	**CHROMATIN-**	86	194	29	69
	**CLG+**	24	1	4	0
	**CLG-**	179	217	54	71
	**CLM+**	18	8	5	2
	**CLM-**	185	209	53	69
	**DNA+**	61	1	19	0
	**DNA-**	118	217	39	71
	**ENA+**	68	11	26	7
	**ENA-**	135	206	32	64
	**RF+**	7	7	4	2
	**RF-**	172	211	54	69
	**SM+**	10	0	2	0
	**SM-**	195	195	56	71
	**SSA_52+**	41	2	16	0
	**SSA_52-**	146	203	42	71
	**SSA_60+**	53	2	17	0
	**SSA_60-**	135	213	41	71
	**U1_RNP+**	40	5	17	4
	**U1_RNP-**	147	210	41	67
**CYTOKINES**	**BAFF**	158	172	58	76
	**BLC**	105	71	74	89
	**CRP**	104	73	44	56
	**FASL**	105	74	73	89
	**GDF15**	105	74	74	89
	**IL1RII**	105	74	74	89
	**IL1ra**	103	74	74	89
	**IL6**	157	171	57	73

	**IP10**	105	74	74	89
	**MCP2**	105	74	74	89
	**MCP4**	105	74	74	89
	**MIP1B**	99	54	71	86
	**MMP2**	105	73	44	56
	**MMP8**	105	73	74	89
	**TARC**	105	74	74	89
	**TGFb1**	158	172	58	76
	**TNFRI**	105	74	74	89
	**TNFa**	158	172	58	75

Supplementary Table 2. Enrichment results for SLE-DMPs

ID	Description	Gene Ratio	Bg Ratio	pvalue	p.adjust	qvalue	Gene ID	Count
**R-HSA-913531**	Interferon Signaling	32/57	199/10654	2.45E-41	6.80E-39	6.26E-39	MX1	32
**R-HSA-909733**	Interferon alpha/beta signaling	23/57	69/10654	3.42E-37	4.75E-35	4.37E-35	MX1	23
**R-HSA-877300**	Interferon gamma signaling	17/57	92/10654	1.85E-22	1.71E-20	1.58E-20	B2M	17
**R-HSA-1169410**	Antiviral mechanism by IFN-stimulated genes	11/57	80/10654	2.94E-13	2.04E-11	1.88E-11	MX1	11
**R-HSA-983170**	Antigen Presentation: Folding, assembly and peptide loading of class I MHC	07/57	25/10654	3.82E-11	2.13E-09	1.96E-09	B2M	7
**R-HSA-983169**	Class I MHC mediated antigen processing & presentation	15/57	371/10654	5.89E-10	2.73E-08	2.51E-08	B2M	15
**R-HSA-1236977**	Endosomal/Vacuolar pathway	05/57	11/10654	1.65E-09	6.56E-08	6.04E-08	B2M	5
**R-HSA-1169408**	ISG15 antiviral mechanism	08/57	72/10654	3.70E-09	1.29E-07	1.18E-07	MX1	8
**R-HSA-168928**	DDX58/IFIH1-mediated induction of interferon-alpha/beta	08/57	78/10654	7.08E-09	2.19E-07	2.01E-07	NLRC5	8
**R-HSA-1236974**	ER-Phagosome pathway	08/57	83/10654	1.17E-08	3.24E-07	2.98E-07	B2M	8
**R-HSA-1236975**	Antigen processing-Cross presentation	08/57	99/10654	4.74E-08	1.20E-06	1.10E-06	B2M	8
**R-HSA-936440**	Negative regulators of DDX58/IFIH1 signaling	05/57	34/10654	9.06E-07	2.10E-05	1.93E-05	NLRC5	5
**R-HSA-933541**	TRAF6 mediated IRF7 activation	04/57	29/10654	1.58E-05	0.00033848	0.00031144	IRF7	4
**R-HSA-918233**	TRAF3-dependent IRF activation pathway	03/57	14/10654	5.07E-05	0.00100657	0.00092615	IRF7	3
**R-HSA-198933**	Immunoregulatory interactions between a Lymphoid and a non-Lymphoid cell	06/57	132/10654	6.98E-05	0.00129312	0.00118981	IFITM1	6
**R-HSA-197264**	Nicotinamide salvaging	03/57	19/10654	0.0001324	0.0023005	0.0021167	PARP9	3
**R-HSA-983168**	Antigen processing: Ubiquitination & Proteasome degradation	08/57	309/10654	0.00021925	0.00358543	0.00329897	PSMB8	8
**R-HSA-933542**	TRAF6 mediated NF-kB activation	03/57	24/10654	0.00027136	0.00419093	0.0038561	IFIH1	3
**R-HSA-912694**	Regulation of IFNA signaling	03/57	26/10654	0.00034594	0.0050617	0.0046573	USP18	3
**R-HSA-196807**	Nicotinate metabolism	03/57	31/10654	0.00058685	0.00815721	0.00750549	PARP9	3
**R-HSA-1059683**	Interleukin-6 signaling	02/57	11/10654	0.00149963	0.01985219	0.01826612	STAT1	2
**R-HSA-933543**	NF-kB activation through FADD/RIP-1 pathway mediated by caspase-8 and -10	02/57	12/10654	0.00179338	0.02266176	0.02085122	IFIH1	2
**R-HSA-877312**	Regulation of IFNG signaling	02/57	14/10654	0.00245576	0.02968262	0.02731116	STAT1	2

**Hypermethylated results**								
**R-HSA-909733**	Interferon alpha/beta signaling	03/24	69/10654	0.00047736	0.04787189	0.04147605	OASL	3
**R-HSA-1169410**	Antiviral mechanism by IFN-stimulated genes	03/24	80/10654	0.00073649	0.04787189	0.04147605	OASL	3
**R-HSA-877300**	Interferon gamma signaling	03/24	92/10654	0.00110594	0.04792408	0.04152126	OASL	3

Supplementary Table 3. Enrichment results for molecular groups-DMPs

A. Enrichment results for inflammatory-only DMP

ID	Description	Gene Ratio	Bg Ratio	P	P adjusted	qvalue	Count	Gene ID
**Hypomethylated results**								
**R-HSA-909733**	Interferon alpha/beta signaling	18/118	69/10654	1.78E-20	8.37E-18	8.30E-18	18	MX1/IFIT1/IFITM1/RSAD2/IFIT3/OAS2/IRF9/ADAR/BST2/USP18/IRF7/PSMB8/IFIT2/SOCS3/HLA-B/OAS1/ISG15/OASL
**R-HSA-913531**	Interferon Signaling	22/118	199/10654	2.56E-16	6.03E-14	5.98E-14	22	MX1/IFIT1/IFITM1/RSAD2/IFIT3/OAS2/B2M/IRF9/ADAR/BST2/USP18/IRF7/SP100/PSMB8/HERC5/EIF4G1/IFIT2/SOCS3/HLA-B/OAS1/ISG15/OASL
**R-HSA-1169410**	Antiviral mechanism by IFN-stimulated genes	9/118	80/10654	2.21E-07	3.47E-05	3.45E-05	9	MX1/IFIT1/OAS2/USP18/HERC5/EIF4G1/OAS1/ISG15/OASL
**R-HSA-877300**	Interferon gamma signaling	9/118	92/10654	7.42E-07	8.74E-05	8.67E-05	9	OAS2/B2M/IRF9/IRF7/SP100/SOCS3/HLA-B/OAS1/OASL
**R-HSA-1169408**	ISG15 antiviral mechanism	6/118	72/10654	0.00013993	0.01318168	0.01308005	6	MX1/IFIT1/USP18/HERC5/EIF4G1/ISG15
**R-HSA-168928**	DDX58/IFIH1-mediated induction of interferon-alpha/beta	6/118	78/10654	0.00021798	0.01711118	0.01697924	6	NLRC5/IFIH1/NKIRAS2/IRF7/HERC5/ISG15
**R-HSA-936440**	Negative regulators of DDX58/IFIH1 signaling	4/118	34/10654	0.00051302	0.0345187	0.03425255	4	NLRC5/IFIH1/HERC5/ISG15
**Hypermethylated results**								
**R-HSA-6794361**	Neurexins and neuroligins	2-jul	56/10654	0.000560324	0.008965187	0.004718519	2	DLGAP1/NRXN3
**R-HSA-6794362**	Protein-protein interactions at synapses	2-jul	87/10654	0.001347981	0.010783845	0.005675708	2	DLGAP1/NRXN3
**R-HSA-425561**	Sodium/Calcium exchangers	1-jul	13/10654	0.008512578	0.037516244	0.019745391	1	SLC24A3
**R-HSA-8951664**	Neddylation	2-jul	234/10654	0.009379061	0.037516244	0.019745391	2	KLHL22/ZBTB16

B. Enrichment results for interferon-only DMS

ID	Description	Gene Ratio	Bg Ratio	P	P adjusted	qvalue	Count	Gene ID
**Hypomethylated results**								
**R-HSA-913531**	Interferon Signaling	37/83	199/10654	8.56E-43	3.16E-40	2.80E-40	37	MX1/IFITM1/IFIT1/IFIT3/IRF9/RSAD2/IRF7/OAS2/B2M/ADAR/BST2/USP18/SP100/PSMB8/STAT1/ISG15/OAS1/IFIT2/HLA-B/HERC5/TRIM34/TRIM22/HLA-F/OASL/EIF2AK2/TRIM25/TRIM14/HLA-A/SOCS3/OAS3/XAF1/HLA-E/DDX58/IFI35/TRIM21/IFI27/HLA-C
**R-HSA-909733**	Interferon alpha/beta signaling	27/83	69/10654	9.36E-41	1.73E-38	1.53E-38	27	MX1/IFITM1/IFIT1/IFIT3/IRF9/RSAD2/IRF7/OAS2/ADAR/BST2/USP18/PSMB8/STAT1/ISG15/OAS1/IFIT2/HLA-B/HLA-F/OASL/HLA-A/SOCS3/

								OAS3/XAF1/HLA-E/IFI35/IFI27/HLA-C
**R-HSA-877300**	Interferon gamma signaling	20/83	92/10654	3.17E-24	3.89E-22	3.46E-22	20	IRF9/IRF7/OAS2/B2M/SP100/STAT1/OAS1/HLA-B/TRIM34/TRIM22/HLA-F/OASL/TRIM25/TRIM14/HLA-A/SOCS3/OAS3/HLA-E/TRIM21/HLA-C
**R-HSA-1169410**	Antiviral mechanism by IFN-stimulated genes	13/83	80/10654	3.04E-14	2.81E-12	2.49E-12	13	MX1/IFIT1/OAS2/USP18/STAT1/ISG15/OAS1/HERC5/OASL/EIF2AK2/TRIM25/OAS3/DDX58
**R-HSA-983170**	Antigen Presentation: Folding, assembly and peptide loading of class I MHC	8/83	25/10654	9.34E-12	6.89E-10	6.11E-10	8	B2M/TAP1/HLA-B/HLA-F/HLA-A/HLA-E/HLA-C/TAP2
**R-HSA-1236977**	Endosomal/Vacuolar pathway	6/83	11/10654	8.33E-11	5.13E-09	4.55E-09	6	B2M/HLA-B/HLA-F/HLA-A/HLA-E/HLA-C
**R-HSA-1236974**	ER-Phagosome pathway	10/83	83/10654	7.24E-10	3.82E-08	3.39E-08	10	B2M/TAP1/PSMB8/HLA-B/HLA-F/HLA-A/HLA-E/HLA-C/TAP2/PSMB9
**R-HSA-983169**	Class I MHC mediated antigen processing & presentation	17/83	371/10654	2.82E-09	1.30E-07	1.15E-07	17	B2M/TAP1/PSMB8/HLA-B/HERC5/HLA-F/RNF213/HLA-A/SOCS3/HLA-E/TRIM69/TRIM21/HLA-C/HERC6/TAP2/PSMB9/ZNRF2
**R-HSA-1169408**	ISG15 antiviral mechanism	9/83	72/10654	3.88E-09	1.55E-07	1.37E-07	9	MX1/IFIT1/USP18/STAT1/ISG15/HERC5/EIF2AK2/TRIM25/DDX58
**R-HSA-1236975**	Antigen processing-Cross presentation	10/83	99/10654	4.20E-09	1.55E-07	1.37E-07	10	B2M/TAP1/PSMB8/HLA-B/HLA-F/HLA-A/HLA-E/HLA-C/TAP2/PSMB9
**R-HSA-168928**	DDX58/IFIH1-mediated induction of interferon-alpha/beta	9/83	78/10654	8.00E-09	2.68E-07	2.38E-07	9	NLRC5/IRF7/IFIH1/ISG15/HERC5/TRAF2/TRIM25/DDX58/DHX58
**R-HSA-936440**	Negative regulators of DDX58/IFIH1 signaling	6/83	34/10654	2.10E-07	6.47E-06	5.74E-06	6	NLRC5/IFIH1/ISG15/HERC5/TRIM25/DDX58
**R-HSA-933541**	TRAF6 mediated IRF7 activation	5/83	29/10654	2.61E-06	7.39E-05	6.56E-05	5	IRF7/IFIH1/TRAF2/TRIM25/DDX58
**R-HSA-918233**	TRAF3-dependent IRF activation pathway	4/83	14/10654	3.23E-06	8.51E-05	7.55E-05	4	IRF7/IFIH1/TRIM25/DDX58
**R-HSA-933542**	TRAF6 mediated NF-kB activation	4/83	24/10654	3.23E-05	0.000794974	0.000705283	4	IFIH1/TRAF2/TRIM25/DDX58
**R-HSA-9020958**	Interleukin-21 signaling	3/83	10/10654	5.26E-05	0.001213083	0.00107622	3	STAT1/JAK3/STAT4
**R-HSA-198933**	Immunoregulatory interactions between a Lymphoid and a non-Lymphoid cell	7/83	132/10654	7.25E-05	0.001574691	0.00139703	7	IFITM1/B2M/HLA-B/HLA-F/HLA-A/HLA-E/HLA-C
**R-HSA-933543**	NF-kB activation through FADD/RIP-1 pathway mediated by caspase-8 and -10	3/83	12/10654	9.54E-05	0.001954717	0.001734181	3	IFIH1/TRIM25/DDX58
**R-HSA-451927**	Interleukin-2 family signaling	4/83	44/10654	0.000366744	0.00712256	0.006318974	4	STAT1/LGALS9/JAK3/STAT4
**R-HSA-197264**	Nicotinamide salvaging	3/83	19/10654	0.000403757	0.00744932	0.006608871	3	PARP9/PARP14/PARP10

**R-HSA-983168**	Antigen processing: Ubiquitination & Proteasome degradation	9/83	309/10654	0.000645453	0.011341523	0.010061942	9	PSMB8/HERC5/RNF213/SOCS3/TRIM69/TRIM21/HERC6/PSMB9/ZNRF2
**R-HSA-8854691**	Interleukin-20 family signaling	3/83	25/10654	0.000926591	0.015541462	0.013788032	3	STAT1/JAK3/STAT4
**R-HSA-912694**	Regulation of IFNA signaling	3/83	26/10654	0.001041591	0.016710735	0.014825384	3	USP18/STAT1/SOCS3
**R-HSA-1834949**	Cytosolic sensors of pathogen-associated DNA	4/83	63/10654	0.001438617	0.022118744	0.019623247	4	IRF7/TRIM21/IFI16/POLR3GL
**R-HSA-196807**	Nicotinate metabolism	3/83	31/10654	0.001750982	0.025844491	0.022928645	3	PARP9/PARP14/PARP10
**R-HSA-1059683**	Interleukin-6 signaling	2/83	11/10654	0.003151013	0.038867463	0.03448233	2	STAT1/SOCS3
**R-HSA-3371378**	Regulation by c-FLIP	2/83	11/10654	0.003151013	0.038867463	0.03448233	2	TRAF2/TNFSF10
**R-HSA-5218900**	CASP8 activity is inhibited	2/83	11/10654	0.003151013	0.038867463	0.03448233	2	TRAF2/TNFSF10
**R-HSA-69416**	Dimerization of procaspase-8	2/83	11/10654	0.003151013	0.038867463	0.03448233	2	TRAF2/TNFSF10
**R-HSA-5689896**	Ovarian tumor domain proteases	3/83	38/10654	0.003159956	0.038867463	0.03448233	3	IFIH1/TRIM25/DDX58
**R-HSA-8984722**	Interleukin-35 Signalling	2/83	12/10654	0.003762145	0.044781657	0.039729269	2	STAT1/STAT4
**Hypermethylated resutls**								
**R-HSA-512988**	Interleukin-3, Interleukin-5 and GM-CSF signaling	6/113	48/10654	1.06E-05	0.005020939	0.004747721	6	PIK3CD/SYK/RAPGEF1/GAB2/INPP5D/IL5RA
**R-HSA-451927**	Interleukin-2 family signaling	5/113	44/10654	9.59E-05	0.022632751	0.021401174	5	PIK3CD/SYK/GAB2/INPP5D/IL5RA
**R-HSA-912526**	Interleukin receptor SHC signaling	4/113	27/10654	0.000174424	0.027442711	0.025949397	4	PIK3CD/GAB2/INPP5D/IL5RA

Supplementary Table 4. Enrichment results for autoantibody-DMPs

A. Enrichment results for chromatin-only DMS

ID	Description	Gene Ratio	Bg Ratio	P	P adjusted	qvalue	Gene ID	Count
**Hypomethylated results**								
**R-HSA-909733**	Interferon alpha/beta signaling	1/1	69/10654	0.00647644	0.01942932	NA	BST2	1
**R-HSA-913531**	Interferon Signaling	1/1	199/10654	0.01867843	0.02801765	NA	BST2	1
**R-HSA-6798695**	Neutrophil degranulation	1/1	480/10654	0.0450535	0.0450535	NA	BST2	1

B. Enrichment results for DNA-only DMS

ID	Description	Gene Ratio	Bg Ratio	P	P adjusted	qvalue	Gene ID	Count
**R-HSA-913531**	Interferon Signaling	8/12	199/10654	5.98E-12	1.55E-10	6.92E-11	MX1	8
**R-HSA-909733**	Interferon alpha/beta signaling	6/12	69/10654	5.30E-11	6.89E-10	3.07E-10	MX1	6
**R-HSA-1169408**	ISG15 antiviral mechanism	2/12	72/10654	0.00284527	0.0227504	0.01013176	MX1	2
**R-HSA-1169410**	Antiviral mechanism by IFN-stimulated genes	2/12	80/10654	0.00350006	0.0227504	0.01013176	MX1	2
**R-HSA-983168**	Antigen processing: Ubiquitination & Proteasome degradation	3/12	309/10654	0.00437521	0.0227511	0.01013207	TRIM21	3
**R-HSA-983169**	Class I MHC mediated antigen processing & presentation	3/12	371/10654	0.00728963	0.03158841	0.01406771	TRIM21	3
**R-HSA-73614**	Pyrimidine salvage	1/12	11/10654	0.01232593	0.04367847	0.01945195	TYMP	1
**R-HSA-73621**	Pyrimidine catabolism	1/12	12/10654	0.01343953	0.04367847	0.01945195	TYMP	1
**R-HSA-3134975**	Regulation of innate immune responses to cytosolic DNA	1/12	15/10654	0.01677344	0.04649436	0.02070599	TRIM21	1
**R-HSA-1834941**	STING mediated induction of host immune responses	1/12	16/10654	0.01788245	0.04649436	0.02070599	TRIM21	1

Supplementary Table 5. SLE-DMPs target drugs

Gene name	Drug_name	Status	Sources
**JAK3**	R-333	Phase II Clinical Trial	Informa
**SYK**	cevidoplenib	Phase I Clinical Trial	Informa
**SYK**	fostamatinib disodium	Phase I Clinical Trial	Informa
**SYK**	HMPL-523	Phase I Clinical Trial	Informa
**SYK**	PRT2607	Phase I Clinical Trial	Informa
**SYK**	R-333	Phase II Clinical Trial	Informa
**PIK3CD**	duvelisib	Phase I Clinical Trial	Informa
**BCL2**	venetoclax	Phase I Clinical Trial	Informa
**BTLA**	LY-3361237	Phase I Clinical Trial	Informa
**GPD2**	METFORMIN	Phase IV Clinical Trial	Open Targets
**JAK3**	UPADACITINIB	Phase II Clinical Trial	Open Targets
**JAK3**	TOFACITINIB	Phase I Clinical Trial	Open Targets
**JAK3**	R-333	Phase II Clinical Trial	Open Targets
**NDUFS8**	METFORMIN	Phase IV Clinical Trial	Open Targets
**SYK**	R-333	Phase II Clinical Trial	Open Targets
**SYK**	FOSTAMATINIB DISODIUM	Phase II Clinical Trial	Open Targets
**SYK**	FOSTAMATINIB	Phase II Clinical Trial	Open Targets
**VDR**	CHOLECALCIFEROL	Phase II Clinical Trial	Open Targets
**VDR**	ALFACALCIDOL	Phase IV Clinical Trial	Open Targets
**VDR**	CALCITRIOL	Phase II Clinical Trial	Open Targets

Supplementary Note 1. PRECISESADS Clinical Consortium

Lorenzo Beretta[9], Barbara Vigone[9], Jacques-Olivier Pers[10], Alain Saraux[10], Valérie Devauchelle-Pensec[10], Divi Cornec[10], Sandrine Jousse-Joulin[10], Bernard Lauwerys[11], Julie Ducreux[11], Anne-Lise Maudoux[11], Carlos Vasconcelos[12], Ana Tavares[12], Esmeralda Neves[12], Raquel Faria[12], Mariana Brandão[12], Ana Campar[12], António Marinho[12], Fátima Farinha[12], Isabel Almeida[12], Miguel Angel Gonzalez-Gay Mantecón[13], Ricardo Blanco Alonso[13], Alfonso Corrales Martínez[13], Ricard Cervera[14], Ignasi Rodríguez-Pintó[14], Gerard Espinosa[14], Rik Lories[15], Ellen De Langhe[15], Nicolas Hunzelmann[16], Doreen Belz[16], Torsten Witte[17], Niklas Baerlecken[17], Georg Stummvoll[18], Michael Zauner[18], Michaela Lehner[18], Eduardo Collantes[19], Rafaela Ortega Castro[19], Ma Angeles Aguirre-Zamorano[19], Alejandro Escudero-Contreras[19], Ma Carmen Castro-Villegas[20], Norberto Ortego[20], María Concepción Fernández Roldán[20], Enrique Raya[21], Inmaculada Jiménez Moleón[21], Enrique de Ramon[22], Isabel Díaz Quintero[22], Pier Luigi Meroni[23], Maria Gerosa[23], Tommaso Schioppo[23], Carolina Artusi[23], Carlo Chizzolini[24], Aleksandra Zuber[24], Donatienne Wynar[24], Laszló Kovács[25], Attila Balog[25], Magdolna Deák[25], Márta Bocskai[25], Sonja Dulic[25], Gabriella Kádár[25], Falk Hiepe[26], Velia Gerl[26], Silvia Thiel[26], Manuel Rodriguez Maresca[27], Antonio López-Berrio[27], Rocío Aguilar-Quesada[27] & Héctor Navarro-Linares[27]

[9]Referral Center for Systemic Autoimmune Diseases, Fondazione IRCCS Ca' Granda Ospedale Maggiore Policlinico di Milano, Milano, Italy. [10]Centre Hospitalier Universitaire de Brest, Hospital de la Cavale Blanche, Brest, France. [11]Pôle de pathologies rhumatismales systémiques et inflammatoires, Institut de Recherche Expérimentale et Clinique, Université catholique de Louvain, Brussels, Belgium. [12]Centro Hospitalar do Porto, Porto, Portugal. [13]Servicio Cantabro de Salud, Hospital Universitario Marqués de Valdecilla, Santander, Spain. [14]Hospital Clinic I Provicia, Institut d'Investigacions Biomèdiques August Pi i Sunyer, Barcelona, Spain. [15]Katholieke Universiteit Leuven, Leuven, Belgium. [16]Klinikum der Universitaet zu Koeln, Cologne, Germany. [17]Medizinische Hochschule Hannover, Hannover, Germany. [18]Medical University Vienna, Vienna, Austria. [19]Servicio Andaluz de Salud, Hospital Universitario Reina Sofía Córdoba, Córdoba, Spain. [20]Servicio Andaluz de Salud, Complejo hospitalario Universitario de Granada (Hospital Universitario San Cecilio), Granada, Spain. [21]Servicio Andaluz de Salud, Complejo hospitalario Universitario de Granada (Hospital Virgen de las Nieves), Granada, Spain. [22]Servicio Andaluz de Salud, Hospital Regional Universitario de Málaga, Málaga, Spain. [23]Università degli studi di Milano, Milan, Italy. [24]Hospitaux Universitaires de Genève, Genève, Switzerland. [25]University of Szeged, Szeged, Hungary. [26]Charite, Berlin, Germany. [27]Andalusian Public Health System Biobank, Granada, Spain.

Supplementary Note 2. PRECISESADS Flow Cytometry Study Group

Montserrat Alvarez[28], Damiana Alvarez-Errico[29], Nancy Azevedo[30], Nuria Barbarroja[31,32], Anne Buttgereit[33], Qingyu Cheng[34], Carlo Chizzolini[28], Jonathan Cremer[35], Aurélie De Groof[36], Ellen De Langhe[37], Julie Ducreux[36], Aleksandra Dufour[28], Velia Gerl[34], Maria Hernandez-Fuentes[38], Laleh Khodadadi[34], Katja Kniesch[39], Tianlu Li[34], Chary Lopez-Pedrera[36], Zuzanna Makowska[33], Concepción Marañón[41], Brian Muchmore[41], Esmeralda Neves[30], Bénédicte Rouvière[42], Quentin Simon[42], Elena Trombetta[40], NievesVarela[39] &TorstenWitte[39]
[28]Immunology and Allergy, University Hospital and School of Medicine, Geneva, Switzerland. [29]Chromatin and Disease Group, Bellvitge Biomedical Research Institute (IDIBELL), Barcelona, Spain. [30]Serviço de Imunologia EX-CICAP, Centro Hospitalar e Universitário do Porto, Porto, Portugal. [31]IMIBIC, Reina Sofia Hospital, University of Cordoba, Córdoba, Spain. [32]Bayer AG, Berlin, Germany. [33]Pharmaceuticals Division, Bayer Pharma, Berlin, Germany. [34]Department of Rheumatology and Clinical Immunology, Charité University Hospital, Berlin, Germany. [35]Department of Microbiology and Immunology, Laboratory of Clinical Immunology, KU Leuven, Leuven, Belgium. [36]Pôle de Pathologies Rhumatismales Inflammatoires et Systémiques, Institut de Recherche Expérimentale et Clinique, Université Catholique de Louvain, Brussels, Belgium. [37]University Hospitals Leuven and Skeletal Biology and Engineering Research Center, KU Leuven, Leuven, Belgium. [38]UCB, Slough, UK. [39]Klinik für Immunologie Und Rheumatologie, Medical University Hannover, Hannover, Germany. [40]Laboratorio di Analisi Chimico Cliniche e Microbiologia - Servizio di Citofluorimetria, Fondazione IRCCS Ca' Granda Ospedale Maggiore Policlinico di Milano, Milan, Italy. [41]GENYO, Center for Genomics and Oncological Research Pfizer/University of Granada/Andalusian Regional Government, Granada, Spain. [42]NSERM, UMR1227, CHRU Morvan, Lymphocytes B et Autoimmunité, University of Brest, BP 824, Brest, France.

Supplementary Note 3. List of Approved Local Ethical Committees

- Referral Center for Systemic Autoimmune Diseases, Fondazione IRCCS Ca' Granda Ospedale Maggiore Policlinico di Milano, Comitato Etico Italy.
- Centre Hospitalier Universitaire de Brest, Hospital de la Cavale Blanche, Avenue Tanguy Prigent 29,609, Brest, France. Comite de Protection des Personnes Ouest VI.
- Pôle de pathologies rhumatismales systémiques et inflammatoires, Institut de Recherche Expérimentale et Clinique, Université catholique de Louvain, Brussels, Belgium. Comité d`Èthique Hospitalo-Facultaire.
- Centro Hospitalar do Porto, Portugal. Comissao de ética para a Saude – CES do CHP.
- Servicio Cantabro de Salud, Hospital Universitario Marqués de Valdecilla, Santander, Spain. Comite ético de investigacion clinical de Cantabria. IDIVAL.
- Hospital Clinic I Provicia, Institut d'Investigacions Biomèdiques August Pi i Sunyer, Barcelona, Spain. Comité Ética de Investigación Clínica del Hospital Clínic de Barcelona. HOSPITAL CLíNIC DE BARCELONA.
- Katholieke Universiteit Leuven, Belgium. Commissie Medische Ethiek UZ KU Leuven /Onderzoek.
- Klinikum der Universitaet zu Koeln, Cologne, Germany. Geschaftsstelle Ethikkommission
- Medizinische Hochschule Hannover, Germany. Ethikkommission.
- Medical University Vienna, Vienna, Austria. Ethik Kommission. Borschkegasse.
- Servicio Andaluz de Salud, Hospital Universitario Reina Sofía Córdoba, Spain. Comité de Ética e la Investigación de Centro de Granada (CEI – Granada).
- Servicio Andaluz de Salud, Complejo hospitalario Universitario de Granada (Hospital Universitario San Cecilio), Spain. Comité de Ética e la Investigación de Centro de Granada (CEI – Granada).
- Servicio Andaluz de Salud, Complejo hospitalario Universitario de Granada (Hospital Virgen de las Nieves), Spain. Comité de Ética e la Investigación de Centro de Granada (CEI – Granada).
- Servicio Andaluz de Salud, Hospital Regional Universitario de Málaga, Spain. Comité de Ética e la Investigación de Centro de Granada (CEI – Granada).
- Università degli studi di Milano, Milan, Italy. Policlinico di Milano, Comitato Etico Italy.
- Hospitaux Universitaires de Genève, Switzerland. DEAS –Commission Cantonale d`´ethique de la recherche Hopitaux universitaires de Geneve.
- University of Szeged, Szeged, Hungary. Csongrad Megyei Kormanyhivatal.
- Charite, Berlin, Germany. Ethikkommission.
- Andalusian Public Health System Biobank, Granada, Spain.
- Comité de Ética e la Investigación de Centro de Granada (CEI – Granada).

Supplementary Methods. Details of platforms and date sources used for scores of methylated genes

1. *The Open Targets (OT) platform* (https://www.opentargets.org/) is a public-private research partnership aimed at integration of multiple target-disease linkage evidence and the development of exploratory methods to facilitate drug target selection and validation. A broad range of target-disease association features were aggregated in OT from public domain information sources, specifically genetic association, somatic mutation, pathway biology, transcriptomics, text mining, animal model, and known drug target status.

2. *Informa* is one of the largest databases for global clinical trials. It aggregates drug, drug targets and clinical trial information from over 40,000 data sources in the public domain, including company press releases, government drug and trial databases (e.g., Drugs@FDA and ClinicalTrials.gov), and scientific conferences and publications. The full dataset can be accessed online at https://citeline.informa.com/.

3. *DisGeNET* (http://www.disgenet.org) consists of large collections of gene to human disease and phenotype associations. The database collects the information from multiple sources such as expert curated repositories, GWAS catalogues, animal models and scientific literature. A gene-disease association (GDA) score (ranging from 0 to 1) is reported as an estimate of the strength of each association.

4. *UK Biobank(UKBB).* Genetic associations were retrieved from GWAS summary statistics from UKBB (https://www.ukbiobank.ac.uk/). A p-value threshold of 5 × 10-8 was used to identify significant genetic variant associations to SLE trait.

5. *PPI network* was from STRING database (https://string-db.org/, version11). The STRING database is one of the most comprehensive protein to protein interaction network with predicted and known interactions. Each edge is given a weight to identify the degree of confidence. In order to generate a reliable, high-trust level network reference in this work, we selected interactions with confidence score greater than 0.7 defined by STRING. After data preprocessing, we reconstructed our global protein-protein interaction network with 16,795 nodes and 252,013 edges.
